# Role of spontaneous and sensory orexin network dynamics in rapid locomotion initiation

**DOI:** 10.1016/j.pneurobio.2020.101771

**Published:** 2020-04

**Authors:** Mahesh M. Karnani, Cornelia Schöne, Edward F. Bracey, J. Antonio González, Paulius Viskaitis, Han-Tao Li, Antoine Adamantidis, Denis Burdakov

**Affiliations:** a Department of Health Sciences and Technology, ETH Zürich, Zürich, Switzerland; b The Francis Crick Institute, London, UK; c Institute of Psychiatry, Psychology & Neuroscience, King’s College London, UK; d Systems Neuroscience, University of Göttingen, Germany; e The Rowett Institute, School of Medicine, Medical Sciences and Nutrition, University of Aberdeen, UK; f Department of Neurology, Inselspital, University of Bern, Switzerland; g Neuroscience Center Zürich (ZNZ), ETH Zürich and University of Zürich, Zürich, Switzerland; h Department of Biomedical Research, University of Bern, Switzerland

**Keywords:** Hypothalamus, orexin/hypocretin neurons, Locomotion, Sensorimotor processing, Ensemble imaging

## Abstract

Appropriate motor control is critical for normal life, and requires hypothalamic hypocretin/orexin neurons (HONs). HONs are slowly regulated by nutrients, but also display rapid (subsecond) activity fluctuations *in vivo*. The necessity of these activity bursts for sensorimotor control and their roles in specific phases of movement are unknown. Here we show that temporally-restricted optosilencing of spontaneous or sensory-evoked HON bursts disrupts locomotion initiation, but does not affect ongoing locomotion. Conversely, HON optostimulation initiates locomotion with subsecond delays in a frequency-dependent manner. Using 2-photon volumetric imaging of activity of >300 HONs during sensory stimulation and self-initiated locomotion, we identify several locomotion-related HON subtypes, which distinctly predict the probability of imminent locomotion initiation, display distinct sensory responses, and are differentially modulated by food deprivation. By causally linking HON bursts to locomotion initiation, these findings reveal the sensorimotor importance of rapid spontaneous and evoked fluctuations in HON ensemble activity.

## Highlights

• Hypocretin/orexin neuron (HON) activation correlates with and is causally linked to locomotion initiation.
• HONs constitute 5 subtypes based on their dynamics before and during locomotion.
• About 2/3 HONs display excitatory sensory responses.
• HON sensory responses mediate a rapid sensory-locomotor transformation.

## Graphical abstract

**Figure:**
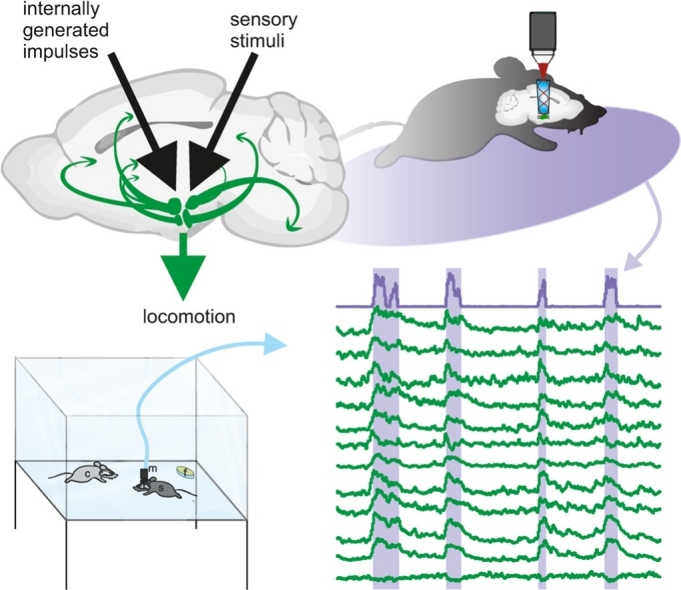

## 1 Introduction

A core function of the nervous system is to generate movements that enable exploration of the environment and rapidly link sensory input to action. The importance of the neocortex, and other classical sensory and motor brain regions for subsecond sensorimotor transformations is well established (Crochet et al., 2019; Svoboda and Li, 2018). In contrast, evolutionarily older regions like the hypothalamus have not been linked to ongoing sensorimotor decision-making, apart from slow modulation of background processes like arousal. However, recent studies show that hypothalamic hypocretin/orexin neurons (HONs) (Tyree et al., 2018) change their activity on a subsecond timescale, including rapid responses to external sensory stimulation and activity associated with movement (González et al., 2016a; Hassani et al., 2016; Inutsuka et al., 2016; Lee et al., 2005; Mileykovskiy et al., 2005; Takahashi et al., 2008). These observations contrast with traditional views of these cells as sensors of slowly-changing variables such as hormones and nutrients that control arousal and locomotion on longer timescales (Adamantidis et al., 2007; Sakurai et al., 1998; Stanojlovic et al., 2019; Yamanaka et al., 2003). Furthermore, chronic HON deletion results in sensorially inappropriate transitions between sleep and wakefulness, and tonic orexin peptide delivery inhibits these sensorimotor deficits (Kaushik et al., 2018; Mieda et al., 2004), while acute inhibition has no immediate effect on wakefulness during the active period of mice (Tsunematsu et al., 2011, 2013). For a slow output it would be unnecessary to constantly update hypothalamic neurons based on sensory information. If HONs mediate slow/modulatory movement control, why does their activity change on a subsecond timescale? The answer to this question is not clear from above correlative studies, and requires subsecond quantification of movement combined with temporally-controlled targeted disruption of underlying hypothalamic signals, which remains unstudied.

Unit recordings (Hassani et al., 2016; Lee et al., 2005; Mileykovskiy et al., 2005; Takahashi et al., 2008) and averaged population recordings with fiber photometry (González et al., 2016a; Inutsuka et al., 2016) have shown that HONs respond to sensory stimuli within tens of milliseconds, and, in separate experiments, that their activity correlates with muscle activation. Because phasic activity of HON ensembles has not been recorded before at single-cell spatial resolution, it is unclear what proportion of HONs are responsive, and how this activity is coordinated between them. Furthermore, the physiological significance of their sensory responses is unclear. We therefore set out to assess the behavioral correlates of phasic HON ensemble activity, and how rapidly they can control behavior in response to sensory input.

Here, using cellular-resolution calcium imaging of >300 HONs during behavior, we were able to distinguish several HON types based on their natural activity profiles during self-initiated and sensory-evoked locomotor events in behaving mice. We quantified their distinct abilities and predicted future locomotion using machine learning. Using optogenetic manipulation of HONs time-locked to specific phases of locomotion or sensory stimulation, we found rapid functional coupling between HON activity and locomotion initiation.

## 2 Results

### 2.1 Phasic activity in HONs is correlated with locomotion across diverse behaviors

**Fig. 1:**
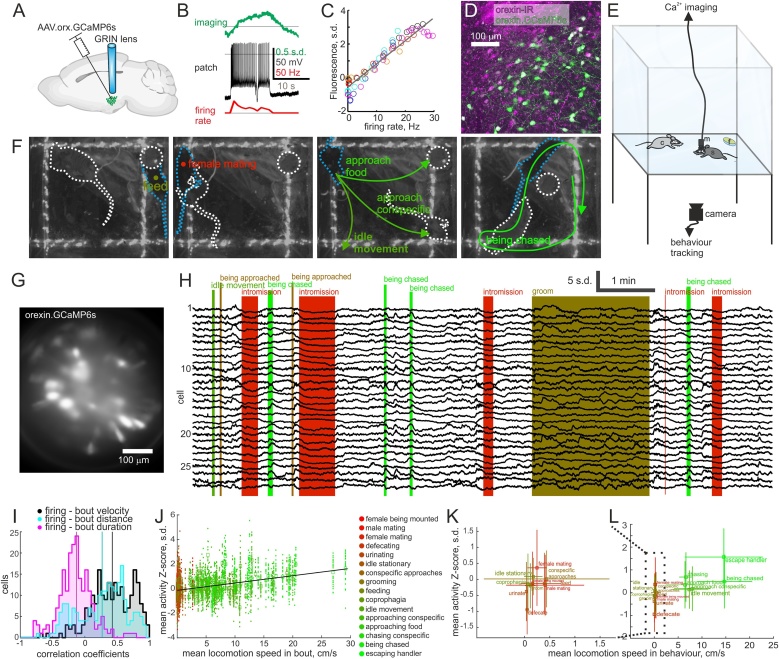
HON Ca^2+^ imaging in freely moving socializing animals. A, A virus was used to drive GCaMP6s expression under the orexin promoter, such that only HONs express GCaMP6s, and GRIN lenses were implanted for optical access to the lateral hypothalamus. B, An HON in a brain slice was imaged during a whole cell patch recording. Ca^2+^-imaging (green, Z-scored ΔF/F0), electrophysiology (black) and firing rate (red) example data shown in full in Figure S1. C, Combined data across 6 recordings (each recording in a different colour) showing linear relation between maximal fluorescence and firing rate during each 10 s current step. R^2^ of linear fit is 0.91. D, Representative micrograph showing orexin immuno-reactivity (IR) and GCaMP6s in a coronal section. E, Schematic of freely-moving *in vivo* paradigm where HONs were recorded with miniature endoscope (m) in an arena with a conspecific (c) and food pod (f) while behavior was tracked from below through the transparent floor. F, Representative behavior video frames showing a female subject (black fur, blue dashed outline) feeding (left), mating (second from left), 3 different medium speed movements (second from right) and being chased (right) by a male conspecific (brown fur, white dashed outline). G, Representative average time projection of Ca^2+^-imaging data through the cylindrical GRIN lens. H, Representative time series of z-scored fluorescence (ΔF/F_0_) of 28 HONs across various behavioral epochs. I, Histogram of Pearson correlation coefficients across all recorded cells (mean correlation with bout speed 0.43 ± 0.02; with distance 0.27 ± 0.03; with duration −0.14 ± 0.02; each distribution significantly different from the others, P < 10^−17^ with paired Student’s *t*-test; 207 cells across 12 recording sessions from 5 animals, 2 male, 3 female). J, Mean Ca^2+^-activity for each cell during each behavior bout (points) and their average (squares) coloured by the behavior class (colour code from red to green follows roughly movement speed in behavior) plotted as a function of mean locomotion speed in behavior bout (249 bouts, 5873 cell/bout pairs). Black line is linear fit to bout average data (squares) with R^2^ = 0.21 and P < 10^−13^. K and L, Same data as in J grouped into behavior classes and plotted as mean ± s.d.

The behavioral output of HONs is often studied in a framework where emphasis is placed on modulation of long-lasting states or regulation of ‘background’ homeostatic variables. If HONs only regulate output variables slowly, they would not need to undergo rapid activity changes on a subsecond timescale and manipulating their activity should not affect processes on the subsecond timescale. In order to probe how fast HON ensemble activity is modulated, we set out to record HONs in awake behaving mice. We recorded the activity of HONs through the fluorescence of the GCaMP6s Ca^2+^ sensor (Chen et al., 2013) which was delivered in a vector under the orexin promoter, restricting expression to HONs (GCaMP6s was expressed with 97.4 ± 1.0 % specificity and 65.8 ± 3.7 % penetrance in HONs, cell counts in Methods)(González et al., 2016b). Simultaneous electrical and optical recordings in brain slices showed that GCaMP6s fluorescence faithfully and rapidly reported spiking activity of HONs (Figs. 1A–D, S1). Using implanted graded refractive index (GRIN, Figs. 1A, S2) lenses and a miniature fluorescence microscope mounted on the skull, we then recorded activity of individual HONs in freely behaving mice as they navigated a familiar 24 × 24 cm arena that contained a food pod and an opposite sex conspecific (Fig. 1E–G). Consistent with previous recordings of single HONs (Mileykovskiy et al., 2005), it was immediately evident that HON ensembles undergo rapid activity modulation, time-locked to natural behaviors lasting only a few seconds (Fig. 1H).

We categorized behaviors into 16 classes that were clearly distinguishable with the camera angle below the animals. Behavior bouts were rapid, lasting on average 11.2 ± 6.6 s (behavior, mean duration ± s.d., number of bouts: female being mounted (without intromission, and not leading to it), 3.0 ± 2.0 s, 11; male mating (intromission), 13.8 ± 7.7 s, 15; female mating (intromission), 32.6 ± 29.4 s, 4; defecating, 6.7 ± 1.6 s, 5; urinating, 3.6 ± 1.3 s, 3; idle stationary (no activity or goal detected), 22.5 ± 25.0 s, 19; conspecific approaches, 2.1 ± 2.8 s, 32; grooming, 38.4 ± 39.6 s, 19; feeding, 46.5 ± 38.8 s, 13; coprophagia, 19.4 s, 1; idle movement (self-initiated locomotion without a detected goal), 4.5 ± 2.9 s, 37; approaching conspecific, 3.0 ± 2.0 s, 43; approaching food, 5.2 ± 4.5 s, 22; chasing conspecific, 7.8 ± 7.1 s, 12; being chased (by conspecific), 6.8 ± 4.2 s, 21; escaping handler, 3.6 ± 3.8 s, 6). Across behavioral classes, activity of 33.3 % of HONs was strongly correlated (Pearson correlation coefficient >0.6) with the speed of locomotion during the behavior, 17.4 % with distance moved, and 1.9 % with duration (Fig. 1I). There was a high similarity between each cell’s speed and distance correlations (linear regression R^2^ = 0.58, P = 10^−40^) indicating that many of the cells code for both speed and distance. Overall, speed was most strongly correlated with HON activity (statistics in figure legends). This correlation was evident across all behavior bouts irrespective of behavior class (Fig. 1J), as well as across averaged metrics within behaviors (Fig. 1K, L).

### 2.2 HONs comprise five locomotion subtypes

**Fig. 2:**
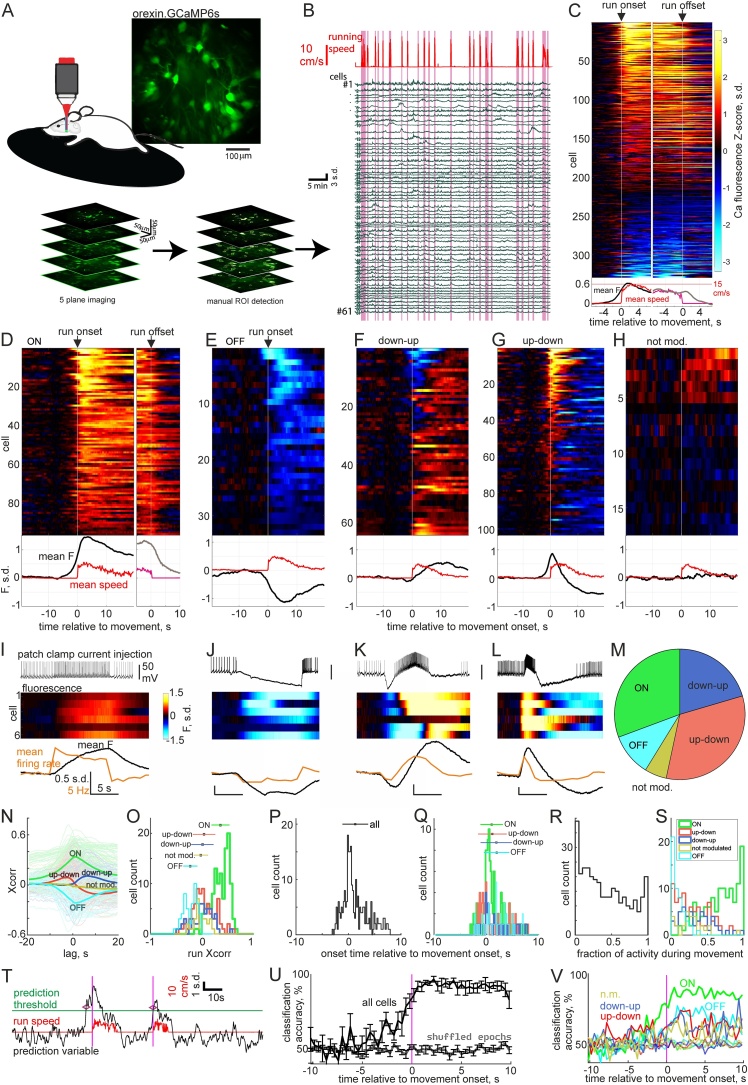
Head-fixed 2-photon imaging of ongoing HON activity in absence of sensory stimuli. A, Schematic of recording paradigm and example average time projection micrograph (above), and example 5-plane volume imaging with manual ROI detection to average fragments of the same cell across planes (below). B, Example fluorescence activity traces from 61 HONs recorded from one volume and running speed measured from rotation speed of treadmill. C, Averaged activity across self-paced locomotion bouts aligned at locomotion start and end (from 329 cells across 7 animals). Mean fluorescence (black) and run speed (red) across all cells plotted below. D–H, Activity traces from C replotted as subtype groups as defined in methods, aligned to movement start, and for B only, also movement end. Scale bar is same as in C. I–L, Data from slice patch clamp recordings combined with Ca-imaging similar to Fig. 1C. Cells were recorded in current clamp and injected current waveforms to mimic expected firing profiles in each subtype in D–G (examples shown on top row of each panel). Z-scored fluorescence traces from each cell in middle raster plot and averaged firing rate (orange) and fluorescence (black) across cells in bottom plot. Scale bars are the same for I–L, except for bottom plots where units for scale bars are the same (5 s.d., 5 Hz and 5 s). M, Proportions of cells in D–H. N, Fluorescence-movement speed cross-correlograms for each cell and averages within subtypes. O, Histogram of fluorescence-movement speed Pearson correlation coefficients. Mean ± s.d. for each subtype plotted at arbitrary heights (ON 0.33 ± 0.17; OFF -0.20 ± 0.13; up-down 0.04 ± 0.20; down-up 0.01 ± 0.20; non mod. −0.02 ± 0.12). P, Histogram of onset time relative to movement start for all cells (mean ± s.d. 1.33 ± 2.40 s). Q, Data from P divided by subtype. Mean ± s.d. for each subtype plotted at arbitrary heights (ON 0.73 ± 1.72 s; OFF 2.03 ± 2.47 s; up-down 1.27 ± 2.77 s; down-up 1.87 ± 2.83 s). R, Fraction of active frames (as defined in methods) that occurred during movement. S, Data from R divided by subtype (ON 0.73 ± 0.23; OFF 0.09 ± 0.08; up-down 0.30 ± 0.23; down-up 0.35 ± 0.22; non mod. 0.28 ± 0.20). T, Prediction variable (black trace) generated from cell fluorescence online plotted against run speed (red). Orange vertical lines denote movement onset, purple horizontal line denotes arbitrary prediction threshold (2 s.d.) and purple diamonds indicate when threshold crossing predicted a run onset. U, Support vector machine classifier accuracy to correctly classify locomotion trials based on all simultaneously recorded HONs (mean ± s.e.m. plotted, n = 6 animals). V, Same analysis as in U broken down by cell type. Error bars omitted for clarity. Dashed curves in U and V are empirical chance values arising from randomly permuted epoch labels.

In order to precisely measure locomotion speed and timing, while recording HON network activity with better spatial resolution using 2-photon imaging, we conducted further studies with a head-fixed apparatus where animals were able to locomote *ad libitum* on a disk treadmill (Fig. 2A). 3-dimensional 2-photon imaging of HON network activity revealed that during self-initiated movement (in a dark room) HONs fell into five categories based on their activity profiles around the running bout. ‘ON cells’ increased firing, ‘OFF cells’ decreased firing, while ‘down-up cells’ and ‘up-down cells’ had a biphasic firing pattern, and a minority of HONs were not significantly modulated during movement (Fig. 2A–H, Movie M1; categorization criteria in Methods). Whole-cell recordings combined with epifluorescence imaging in slices confirmed that this range of fluorescence profiles can arise from firing patterns, and that the same HON is biophysically capable of expressing any activity profile (Fig. 2I–L). The majority of the recorded HONs were up-down (33 %) or ON cells (31 %), which showed initial increases in spiking activity. Down-up (20 %) and OFF (10 %) cells, which initially decreased their activity, and non-modulated cells (6%) made up the remainder (Fig. 2M). This diversity of activity profiles during and around locomotion epochs suggests that both burst and tonic increases and decreases in firing occur in different HONs. The HON subtypes did not show a marked spatial clustering within the imaged volumes (Fig. S3), with all subtypes intermingled within the volume. However, the average of ON cell and down-up cell positions was slightly but significantly more posterior than that of down-up cells (63 and 61 μm respectively, P < 0.01 by Wilcoxon rank sum test). These observations suggest there may be a gradient of HON function along the anteroposterior axis.

Each cell type showed a different cross-correlation with treadmill speed (Fig. 2N, O). While response onsets were scattered through the time period 2.91 s before to 7.78 s after locomotion start, median response onset time was 0.58 s after movement onset. Many ON and up-down cells had onsets before movement started (Fig. 2P, Q). The activity of many ON cells occurred solely during locomotion bouts, whereas many OFF cells were active only outside locomotion (Fig. 2R, S; definitions in Methods). EMG recordings during freely moving orexin fiber photometry also confirmed that HON activity increases before muscle activity (Fig. S4).

These findings suggest that some HONs could be used to predict movement before it starts. We assessed this by dividing long recording sessions into two parts, and using the information from the first part to predict when movement would happen in the second part. From the information in the first part, we identified ON cells that had long movement onset lead times and were active solely during movement. A simple threshold-crossing criterion for activity of these cells could detect 80 ± 4 % of imaging frames with movement, and had a false positive rate of only 11 ± 4 % (n = 7 animals). In our highest quality recordings, the weighted average activity vector of these cells predicted movement onset of 75 % (9/12) of locomotion bouts 1.55 ± 0.36 s before they occurred (Fig. 2T). To formally estimate the predictive power of HON activity without bias, we trained a linear support vector machine classifier on training data from each animal and assessed its accuracy to classify test data as movement or stationary. Trials were selected as all locomotion bouts in a recording session, and an equal number of randomly selected stationary epochs without any running during the 20 s epoch. To minimize bias, we used 8-fold cross-validation and averaged accuracy over 10 repetitions of re-drawn analysis runs. This revealed that HONs could increasingly accurately classify movement trials as movement onset approached, reaching 80.3 ± 5.1 % accuracy in the 800 ms preceding movement onset (significantly higher than empirical chance distribution 49.1 ± 2.0 %, P = 0.0025 by paired *t*-test, n = 6 animals, Fig. 2U). ON cells contained the most predictive information (Fig. 2V), with classification accuracy in the 800 ms before movement onset reaching 78.3 ± 6.0 % (P = 0.011). In contrast, OFF cells (57.9 ± 5.3 %), up-down (62.4 ± 5.1 %), down-up (56.2 ± 8.3 %) and non-modulated cells (55.6 ± 4.5 %) were not significantly different from their empirical chance distributions (P > 0.05). As expected, all cell types showed increased classification accuracy after movement onset (Fig. 2U).

### 2.3 HON activity is causally involved in movement initiation

**Fig. 3:**
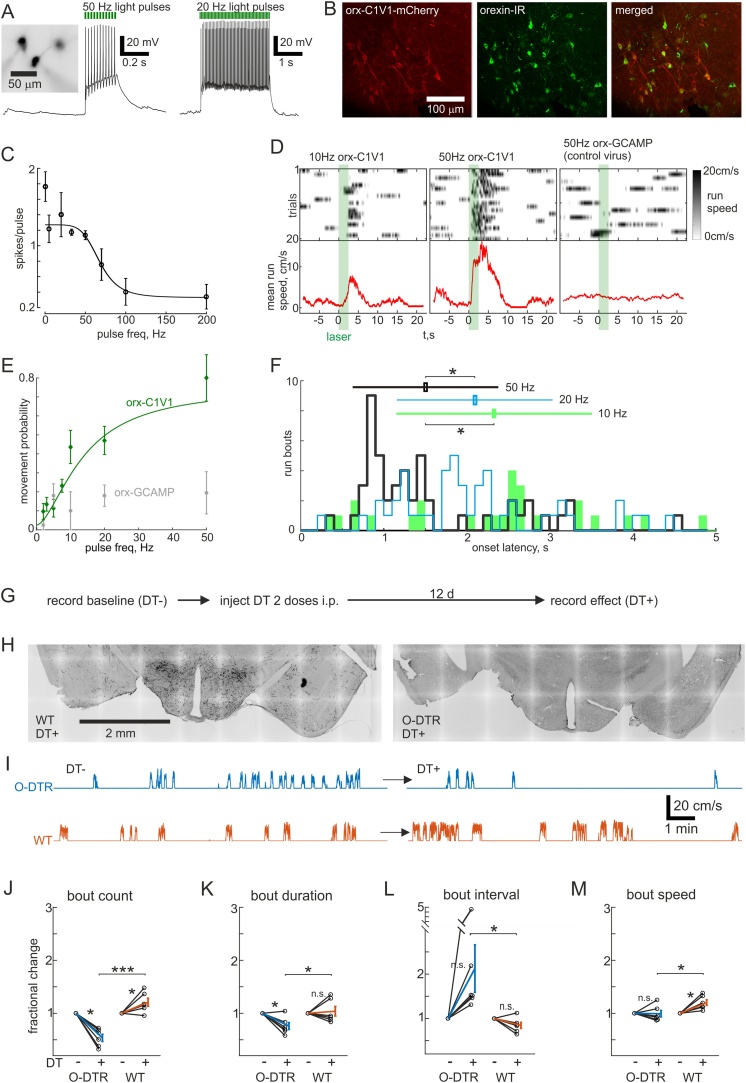
Manipulating HONs controls self-paced movement bidirectionally. A, Micrograph and representative current clamp trace of HON that expresses C1V1. B, Micrographs showing localization of C1V1-mCherry in orexin immunoreactive (IR) cells. C, Tuning curve of action potential fidelity as a function of light pulse frequency (n = 4–6 cells for each data point). Inverse sigmoidal fit explained in methods. D, Treadmill speed across 20 trials and average speed (red) from two head-fixed mice. Mouse with orexin-C1V1 shown with 10 Hz (left) and 50 Hz (middle) light pulses, while mouse with control virus (orx-GCaMP6s) and implanted light fibers shown with 50 Hz light pulses (right). E, Dose response curve of locomotion bout initiation likelihood as a function of light pulse frequency (n = 4–6 animals for each data point). F, Histogram of orexin-C1V1 stimulation induced run bout onset latencies from the onset of a 2.5 s light pulse train at the indicated frequencies. Mean ± s.d. for each frequency plotted at arbitrary heights (10 Hz 2.33 ± 1.18 s; 20 Hz 2.10 ± 0.94 s; 50 Hz 1.50 ± 0.88 s, *P < 0.01 using the Wilcoxon rank sum test). G, Recording schedule for comparing movement in the same animals with and without HONs. H, Representative micrographs of orexin immunoreactivity in wild type (WT) and orexin-diphtheria toxin receptor (O-DTR) brains >12 days after diphtheria toxin injection (DT+). I, Representative treadmill speed recordings from head-fixed O-DTR and WT mice before and after DT administration. J–M, Averaged movement bout statistics normalized to baseline (6 mice/cohort), tested by paired Student’s *t*-test within groups and unpaired across groups (*: P < 0.05, ***: P < 0.0001): J, number of movement bouts during a 16.3 min recording (O-DTR DT + 54 ± 7 %; WT DT + 121 ± 8 %); K, duration of bouts (O-DTR DT + 76 ± 7 %; WT DT + 104 ± 9 %); L, duration of intervals between bouts (O-DTR DT + 213 ± 54 %; WT DT + 84 ± 7 %); M, average speed of bouts (O-DTR DT + 99 ± 6 %; WT DT + 120 ± 5 %).

Since the activity of HONs could predict movement with high temporal resolution, we asked if their activation could elicit locomotion on the same timescales. To probe this, we created a viral construct that directs HONs to express the red-shifted channelrhodopsin variant C1V1 (similar to the GCaMP6s virus, C1V1.mCherry was expressed with 96.0 ± 0.2 % specificity and 54.8 ± 3.4 % penetrance in HONs, see Methods). This allowed HON firing to be controlled using green laser pulses (Fig. 3A–C). Because fidelity of firing decreased at laser pulse frequencies above 50 Hz (Fig. 3C), and previous electrical recordings from a small number of HONs *in vivo* suggest that they do not normally fire above this frequency (Lee et al., 2005; Mileykovskiy et al., 2005; Takahashi et al., 2008), we assessed the effects of 2–50 Hz stimulation on locomotion bouts in mice implanted with bilateral light fibers above LH, and transfected with either orexin-C1V1 or orexin-GCaMP6s (as control), while they were head-fixed on a running wheel. Laser stimulation elicited running in a frequency-dependent manner only in C1V1 expressing mice (Fig. 3D, E). The behavioral effect became evident at about 7.5–10 Hz stimulation (Fig. 3E), which is within the natural instantaneous firing range of HONs at 0–15 Hz (Lee et al., 2005; Mileykovskiy et al., 2005). In addition to increasing the probability of starting a locomotion bout, increasing stimulation frequency decreased the latency from stimulation to locomotion onset (Fig. 3F). Overall, the latencies were distributed around a median of 1.75 s (range 300–4880 ms) from stimulus onset which is consistent with the natural latency from orexin ON and up-down cell activation to self-paced locomotion onset (Fig. 2Q). These results indicate that HON activation leads to rapid initiation of movement.

To assess the role of natural HON activity in self-initiated locomotion, we next performed several loss-of-function experiments. First, we ablated HONs using a mouse line expressing the human diphtheria toxin receptor in HONs (O-DTR) (González et al., 2016b). When these mice were injected with a low dose of diphtheria toxin (DT), their HONs were deleted (3.6 ± 1.8 % remaining, n = 6 animals) within 12 days, while DT-injected wild-type control mice maintained their HONs (Fig. 3G, H). We compared statistics of self-initiated locomotion in the dark before and after DT administration to O-DTR and wild-type mice (Fig. 3I–M). After HON deletion, mice ran significantly fewer bouts than prior to deletion, or than in wild-type controls (Fig. 3J). The bouts were also significantly shorter, by 24 ± 7 % compared to before deletion (Fig. 3K). Additionally, in orexin-neuron-ablated mice stationary intervals between runs were longer and locomotion speed was lower than in wild-type controls (Fig. 3L, M). These results suggest that HONs may regulate self-generated movement initiation, maintenance and speed.

**Fig. 4:**
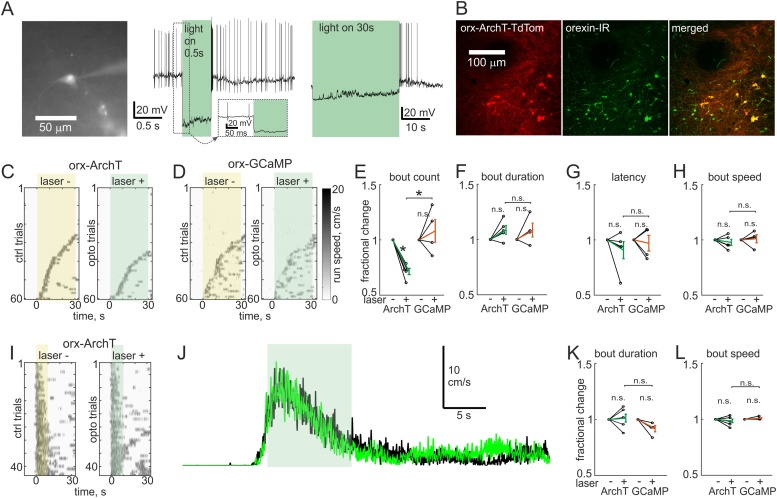
Acute inhibition of HON activation decreases only movement initiation. A, Representative micrograph and current clamp recordings of ArchT expressing HON. B, Representative micrographs of ArchT-TdTomato expression in orexin immunoreactive (IR) neurons. C–D, Example recordings of treadmill speed during self-initiated movement in orexin-ArchT and control (orexin-GCaMP6s with light fiber implants) animals. E–H, Averaged movement bout statistics normalized to baseline from each mouse (5 ArchT mice and 4 control mice), tested by paired Student’s *t*-test (*: P < 0.01). E, number of movement bouts (ArchT 71 ± 3 %; GCaMP 108 ± 10 %); F, duration of bouts (ArchT 109 ± 4 %; GCaMP 109 ± 6 %); G, latency from laser pulse to run bout (ArchT 91 ± 8 %; GCaMP 97 ± 7 %); H, average speed of bouts (ArchT 98 ± 3 %; GCaMP 101 ± 4 %). I, Example recordings of treadmill speed during self-paced running where laser was turned on after movement was detected (on laser + trials). Scale same as in D. J, Average running speed across trials in I, green is from laser + and black from laser- (catch) trials. K–L, Averaged movement bout statistics normalized to baseline from each mouse (5 ArchT mice and 4 control mice), tested by paired Student’s *t*-test (n.s.: P > 0.05). K, duration of bouts (ArchT 101 ± 4 %; GCaMP 92 ± 3 %); L, average speed of bouts (ArchT 97 ± 1 %; GCaMP 101 ± 1%).

The O-DTR method is not selective for HON activity during movement, but ablates all functions of HONs, including their slow indirect effects on movement endurance that may arise due to their role in sympathetic coordination of breathing (Carrive and Kuwaki, 2017). Therefore, to complement this chronic manipulation of HONs, and to selectively disrupt HON activity associated with different phases of movement (Fig. 2), we created a viral construct that directs HONs to express the red-shifted inhibitory opsin ArchT (see Methods). This allowed us to hyperpolarize HONs with green laser light by 26.7 ± 6.7 mV (n = 8 cells, Fig. 4A). With this temporally controllable, reversible loss-of-function approach we could interleave laser-on and laser-off trials and compare to control mice injected with the GCaMP6s virus. To make comparisons across trials consistent, we waited for mice to be immobile for 10 s before either turning the laser on for 30 s to inhibit HONs, or recording a catch trial (laser off). We quantified the movement bouts during this 30 s period. HON inhibition decreased the number of self-initiated locomotion bouts (Fig. 4C–E), but did not affect bout duration, latency to run onset during the assay period, or speed of the run bouts (Fig. 4F–H). In a third loss-of-function experiment we asked what would happen if HONs were inhibited after movement onset, given that some motor system manipulations can interrupt initiated locomotion (Chabrol et al., 2018). However, movement was not disrupted by turning the laser on for 10 s as soon as a run onset was detected (∼0.1–0.5 s delay) when compared with interleaved catch trials (laser off) with at least 30 s intervals between consecutive trials (Fig. 4I, J). There was no effect on duration or speed of runs, suggesting that HON activity after locomotion initiation is not necessary for maintenance of locomotion (Fig. 4K, L). Taken together, these acute optogenetic loss-of-function experiments indicate that the natural activation of HONs initiates self-generated locomotion, but does not modulate the parameters of locomotion bouts once they have started. Unlike optogenetic inhibition, HON deletion decreased run duration and speed (compare Figs. 3K, M and 4 F, H), which suggests that the inhibitory dynamics of some HONs types or indirect tonic functions of HONs may control these parameters (see Discussion).

### 2.4 Overlapping HON ensembles represent locomotion and sensory stimuli

**Fig. 5:**
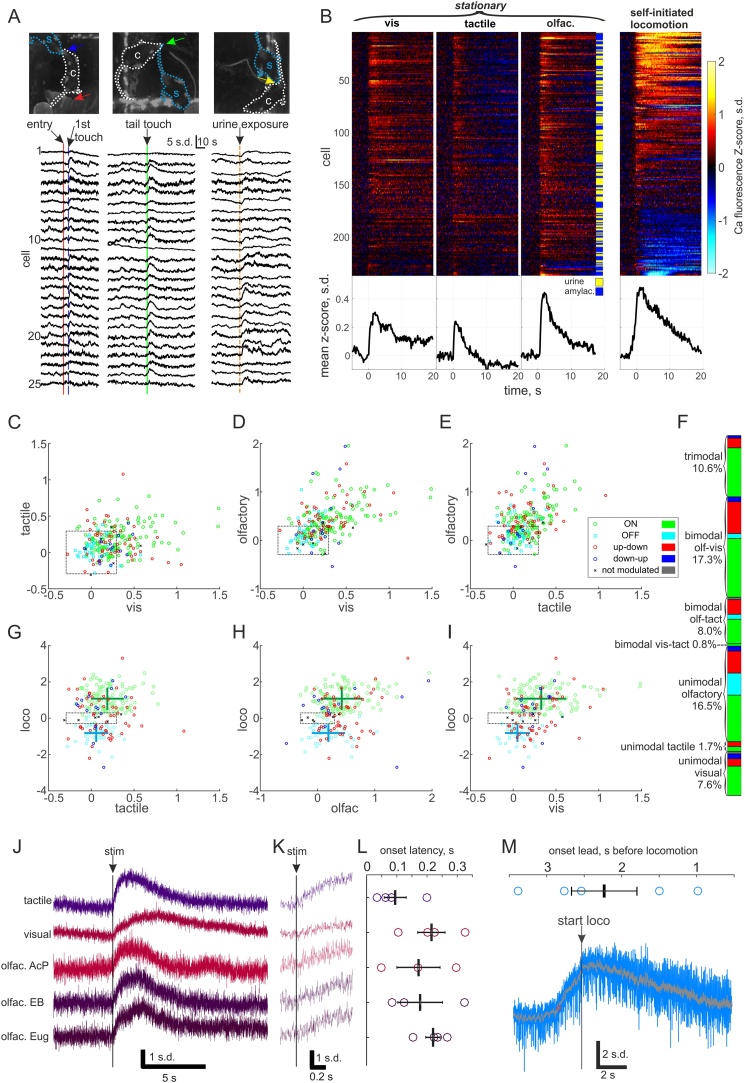
Sensory responses in HONs. A, Example natural sensory responses to social stimuli in HONs recorded with miniature endoscope in freely moving male subject (s, blue dashed outline) while it was in the arena with a female conspecific (c, white dashed outline). Arrows indicate locations of natural stimuli. In left panel conspecific enters arena from handler’s hand (red arrow) and moves to touch snouts with subject (blue arrow). In middle panel conspecific investigates subjects tail, touching it (green arrow) and causing subject to stop feeding and turn around. In right panel subject is investigating conspecific’s rear when conspecific begins to urinate (yellow arrow). B, Head-fixed sensory responses from 237 cells from 4 mice, averaged from 5 to 11 trials where mice remained stationary (three panels from left). Vis = visual, blue LED flash; tactile, 30 psi pressure pulse applied to left side abdomen; olfac. = olfactory, odor pulse (rapid valve switch between empty vial and odor vial with no change in pressure) to snout containing amylacetate (amylac.) or urine pooled from 5 females in different cages. Right panel contains averaged locomotion activity profiles aligned to self-initiated movement onset from each cell from 5 to 22 locomotion bouts outside sensory stimulation trials. Black traces at the bottom of each cell raster are averages across all cells. C–E, Scatter plots of response amplitudes (mean signal from 1 to 4 s after stimulus) of all cells to two modalities in each plot, coloured by the locomotion subtype (legend in E). Dashed black boxes denote the extrema of null-responses (quantified response amplitude to a 77 dB 10 kHz sound stimulus that did not cause a detectable response), *i.e.*, only responses outside the box should be considered meaningful. F, Proportions of responsive cells out of all cells coloured by locomotion subtype and grouped by the number of response modalities. G–I, Scatter plots of response amplitudes and locomotion profile averages (see methods). Legend as in E. Green and cyan crosses denote mean ± s.d. for ON and OFF cell populations respectively (sensory responses: G, ON 0.19 ± 0.19, OFF 0.06 ± 0.14; H, ON 0.42 ± 0.36, OFF 0.19 ± 0.29; I, ON 0.33 ± 0.29, OFF 0.05 ± 0.15) which were significantly different for all modalities (P < 0.001 Wilcoxon rank sum test). J, Fiber photometry responses of the orexin population to tactile, visual and three different olfactory stimuli (AcP, acetophenol; EB, ethylbutyrate; Eug, eugenol). K, Expanded view from J. L, Quantified latency from stimulus onset to response onset tactile (95 ± 36 ms), visual (172 ± 72 ms) and three different olfactory stimuli (acetophenol, 178 ± 74 ms; ethylbutyrate, 220 ± 24 ms; eugenol, 214 ± 45 ms) in averaged traces (5–20 trials) for 4 mice for all stimuli except AcP and EB from 3 mice. M, Onset lead delay from fiber photometry signal increase to movement onset (2230 ± 440 ms) from 11 to 28 locomotion bouts from 5 mice. Blue background trace is unfiltered and black trace is smoothed with a 100-sample moving average.

Given that the main effect of the loss-of-function manipulations was decreased initiation of self-generated movement, we next wanted to inspect the role of HONs in sensory-evoked movement. During freely-moving recordings, we noticed many events that looked like sensory responses, because they coincided with tactile, visual or olfactory stimuli (Fig. 5A). However, it was unclear whether these responses were in fact due to something else, such as movement. Therefore, we presented head-fixed mice with sensory stimuli and only analysed responses when the subject did not locomote during the trial (Fig. 5B). We also recorded the activity profiles of the same cells during self-initiated locomotion and compared responses across modalities and cell types (Fig. 5C–F). All cell types responded with transiently increased activity to visual (blue LED flash), tactile (airpuff to left side abdomen) and olfactory (amylacetate or female urine) stimuli. In all recordings, we estimated the distinction between noise and true response by interleaved presentation of a null stimulus which elicited no responses (see Methods). Sensory responses were significantly different from the null responses (P < 10^−18^; null −0.01 ± 0.01, visual 0.26 ± 0.02, tactile 0.14 ± 0.01; olfactory 0.38 ± 0.03), as was the average locomotor activity (P = 10^−37^; 1.76 ± 0.12; n = 237, paired Student’s T-tests). The null responses also allowed us to delineate thresholds of detection throughout our analysis (null responses fell within dashed black boxes in Fig. 5C–E, G–I). This revealed only two cell-stimulus pairs with inhibitory responses (points on negative side of black boxes in Fig. 5C–E). In total 62.0 % of HONs had excitatory responses to at least one modality, of which 58.5 % responded to more than one modality and 17.0 % to all three (Fig. 5F). Since 57.8 % of responsive cells had visual, 33.3 % tactile and 84.4 % had olfactory responses, the proportions of bimodal and trimodal cells were consistent with chance overlap, suggesting that HONs are not tuned to a particular sensory modality. We also captured some self-initiated locomotion bouts during epochs with no sensory stimulus (Fig. 5B right panel). These data showed that magnitude of peak locomotion modulation did not correlate with the size of sensory responses but the magnitude of sensory responses in ON cells was significantly greater than in OFF cells (P < 0.001 using the Wilcoxon rank sum test, Fig. 5G–I), suggesting that sensory-evoked movements could potentially be elicited through activation of ON cells. Indeed, the ON cell population had the highest proportion of sensory-responsive cells (71 % responded to at least one stimulus modality), while OFF cells had the lowest (33 %), and up-down 67 %, down-up 50 % and non-modulated 33 %.

We used fiber photometry of average signal across GCaMP6s expressing HONs to quantify sensory latencies, because this allowed increasing the sampling rate to 500 Hz. Sensory responses started on average 176 ± 22 ms after stimulus onset (range 34–324 ms), whereas orexin population activity increased above baseline 2230 ± 440 ms before self-initiated locomotion onset (Fig. 5J–M). Although care should be taken when interpreting values <100 ms recorded with the somewhat slow GCaMP6s (see Discussion), this timing is consistent with the possibility that sensory activation of HONs could lead to locomotion.

### 2.5 Phasic HON activity mediates sensorimotor processing

**Fig. 6:**
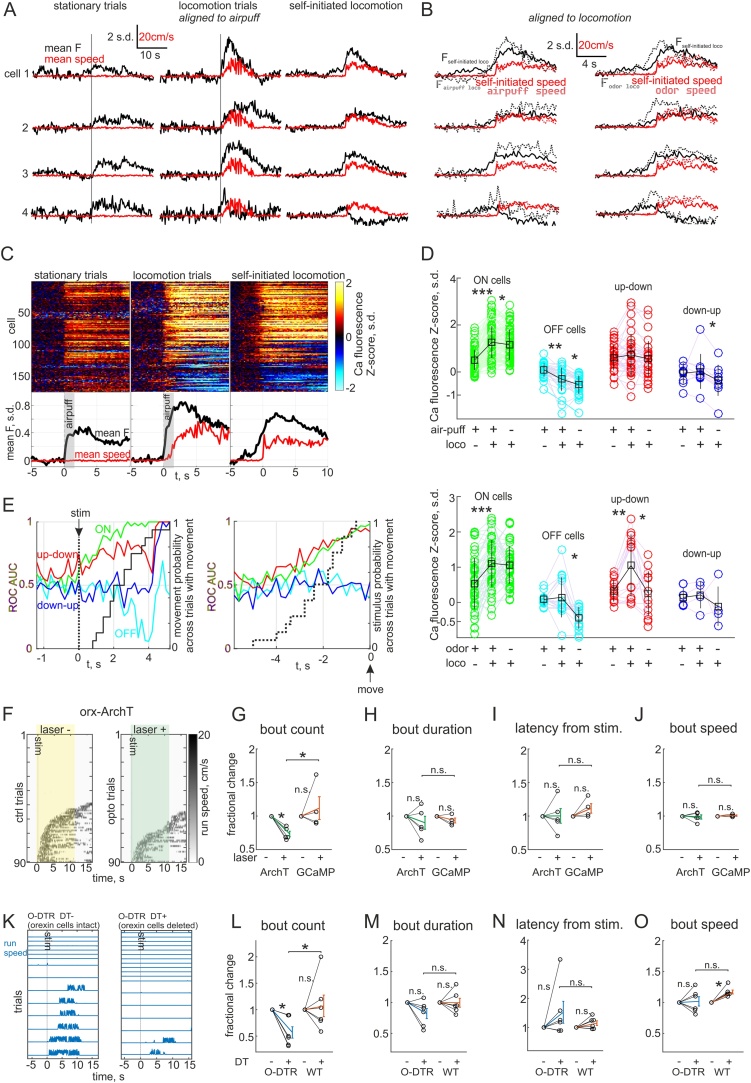
HONs mediate a rapid sensorimotor transformation. A, Example cells responding to tactile stimulus (2 s airpuff 15 psi to base of tail) while mouse was stationary (left) and when the airpuff triggered movement (middle). Also shown activity profiles during self-paced movement without sensory stimuli (right). B, Expanded view of self-paced traces from A (black and red) and stimulus triggered movement (magenta, airpuff on left; social odor on right, consisting of female urine odor presentation for 0.5 s) and response (blue, airpuff on left; social odor on right) aligned to the onset of movement. C, Activity of all recorded cells in response to airpuff while mice were stationary (left, averaged across) and when movement was triggered (middle). Also shown activity profiles during self-paced movement without sensory stimuli (right). Bottom black traces are averages across all cells and red traces movement speed. D, Mean responses measured from an analysis window 1−4 s after sensory stimulus onset (or locomotion onset for self-paced locomotion) in airpuff (above, mean ± s.d. values in black: ON 0.50 ± 0.42, 1.27 ± 0.67, 1.16 ± 0.63; OFF 0.09 ± 0.29, −0.31 ± 0.50, −0.54 ± 0.40; up-down 0.61 ± 0.53, 0.73 ± 0.87, 0.56 ± 0.73; down-up −0.05 ± 0.43, 0.02 ± 0.73, −0.36 ± 0.65) and odor presentation (below, mean ± s.d. values in black: ON 0.53 ± 0.73, 1.11 ± 0.67, 1.06 ± 0.54; OFF 0.08 ± 0.16, 0.14 ± 0.56, −0.44 ± 0.30; up-down 0.33 ± 0.30, 1.05 ± 0.87, 0.26 ± 0.62; down-up 0.14 ± 0.25, 0.21 ± 0.37, −0.11 ± 0.55). * P < 0.025, ** P < 0.001, *** P < 10^−8^. E, Evolution of area under the curve receiver operating characteristic calculated from cell activity data in airpuff stimulation trials where locomotion had not yet started (left panel). Black continuous plot is the cumulative probability distribution of locomotion onsets from trials with movement (16/48). Right panel is ROC AUC calculated from trials aligned to locomotion onset instead of stimulus. Black dashed curve is the cumulative probability distribution of stimuli across trials with movement. F, Example recordings of treadmill speed during sensory evoked running in head-fixed orexin-ArchT animals with laser turned on 0.5 s before stimulus (left) and without laser (right). G–J, Averaged movement bout statistics normalized to baseline from each mouse (5 ArchT mice and 4 control mice), tested by paired Student’s *t*-test (*: P < 0.05). G, number of movement bouts (ArchT 73 ± 4 %; GCaMP 112 ± 17 %); H, duration of bouts (ArchT 90 ± 10 %; GCaMP 93 ± 4 %); I, latency from sensory stimulus to run bout (ArchT 100 ± 11 %; GCaMP 111 ± 7 %); H, average speed of bouts (ArchT 99 ± 3 %; GCaMP 101 ± 1 %). K, Representative treadmill speed recordings during sensory evoked running from head-fixed O-DTR mice after (left) and before (right) DT administration. L–O, Averaged movement bout statistics normalized to baseline from each cohort (6 mice/cohort), tested by paired Student’s *t*-test (*: P < 0.05). L, number of movement bouts (O-DTR DT + 50 ± 10 %; WT DT + 107 ± 21 %); M, duration of bouts (O-DTR DT + 88 ± 8 %; WT DT + 100 ± 7 %); N, latency from sensory stimulus to run bout (O-DTR DT + 165 ± 44 %; WT DT + 115 ± 8 %); O, average speed of bouts (O-DTR DT + 100 ± 9 %; WT DT + 117 ± 3 %).

We probed HON-mediated sensory-evoked movement further by analysing HON activity during stimulation trials where the stimulus evoked movement (Fig. 6A–C). A gentle airpuff to the base of the tail induced locomotion in 33 % (16/48) of trials, and female urine odor induced locomotion in 25 % (12/48) of trials across four animals. When sensory stimulation evoked locomotion, the overall HON responses were bigger than when mice remained stationary during stimulus presentation (Fig. 6C, D). To compare HON activity during sensory-evoked movement to their activity profiles during self-initiated locomotion, we took the sensory stimulus trials where movement was triggered and aligned them to locomotion onset (Fig. 6B). This showed that time-courses were comparable between the conditions (mean latency from HON activity onset to locomotion onset was 1.2 ± 0.4 s for sensory-evoked and 0.9 ± 0.4 s for self-initiated; P = 0.31 by paired *t*-test, n = 95 ON and up-down cells), while sensory-evoked movement was coupled with higher amplitudes than the activity profiles during self-initiated locomotion. ON cells in particular had significantly larger responses during sensory-evoked movement than during sensory responses while stationary (Fig. 6D), and up-down cells were selectively more active when odor stimuli induced movement. In order to examine objective predictive information about future movement in HON subpopulations, we used receiver operating characteristic (ROC) analysis to assess the evolution of the ROC AUC (area under the curve) predictor in each cell type during sensory stimulation trials. Predictive information increased in ON and up-down cells from sensory stimulation until locomotion onset (Fig. 6E). When trials were aligned to the sensory stimulus, ON cell activity predicted the outcome with 68 % accuracy during the 800 ms following sensory stimulus, while up-down cells performed at 65 %, and down-up (43 %) and OFF cells (48 %) performed near chance (Fig. 6E left panel). When trials were aligned to the locomotion onset, a high accuracy of classification in the 800 ms preceding movement onset was evident for ON (95 %) and up-down cells (93 %), while down-up (47 %) and OFF cells (45 %) performed near chance (Fig. 6E right panel). This shows that an ideal observer could use ON, and, to a lesser extent, up-down cell activity to predict the decision to move after the sensory stimulus.

In order to examine if there is a causal link between HON activation and sensory-evoked movement, we tested whether disrupting HON activation by ArchT-mediated hyperpolarization would block this sensorimotor transformation. When HON-ArchT cells were bilaterally inhibited from 0.05 s before to 12 s after a sensory stimulus, significantly fewer locomotion bouts were elicited than in interleaved control stimulation trials, and this difference was not seen in control virus (orexin-GCaMP) injected mice (Fig. 6F, G). However, HON opto-silencing elicited no change in duration, latency or speed of the sensory-evoked movement (Fig. 6H–J). This indicates that excitatory components of HON responses, for example ON cell activation, are critical for normal sensorimotor transformations. To determine if the initial inhibitory responses in OFF and down-up cells might play a role in sensorimotor transformations, we further inspected the role of all HONs in sensory-evoked movement by deleting them in O-DTR mice. The effect of O-DTR DT + HON deletion was a decrease in occurrence of sensory-evoked movement (Fig. 6K–O), similar to the ArchT experiment. This suggests that the activation rather than inactivation of HON subpopulations accounts for their role in sensory-evoked movement. Overall, these results demonstrate that HONs selectively affect sensory-evoked movement initiation rather than maintenance, reaction time or speed.

### 2.6 HON sensory responses are state dependent

**Fig. 7:**
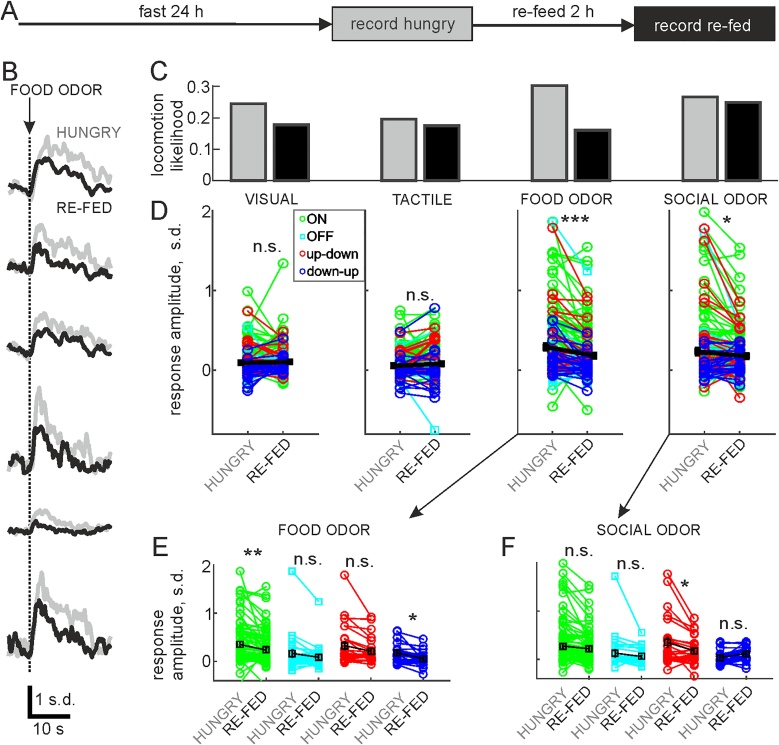
HON sensory responses are state dependent. A, Recording schedule for comparing sensory responses in hungry and re-fed animals. B, Example responses to food odor from six cells in hungry (gray) and re-fed (black) conditions. C, likelihood of initiating a locomotion bout following sensory stimulus across all four mice. D, Mean responses from trials where mice remained stationary, measured from an analysis window 1–4 s after sensory stimulus onset, colour coded by cell type as shown in legend. Overall mean responses in black (hungry response ± s.e.m., re-fed response ± s.e.m., P-value: visual 0.093 ± 0.014, 0.101 ± 0.015, P = 0.49; tactile 0.054 ± 0.012, 0.079 ± 0.016, P = 0.03; food odor 0.291 ± 0.032, 0.181 ± 0.023, P = 10^−8^; social odor 0.233 ± 0.033, 0.174 ± 0.022, P = 0.003). E, Responses to food odor from D by cell type according to legend in D (mean ± s.e.m. values in black: ON 0.34 ± 0.04, 0.24 ± 0.04; OFF 0.15 ± 0.08, 0.08 ± 0.04; up-down 0.32 ± 0.07, 0.21 ± 0.06; down-up 0.18 ± 0.04, 0.06 ± 0.05). F, Responses to social odor from D by cell type according to legend in D (mean ± s.e.m. values in black: ON 0.28 ± 0.04, 0.21 ± 0.04; OFF 0.012 ± 0.07, 0.06 ± 0.04; up-down 0.32 ± 0.10, 0.17 ± 0.06; down-up 0.04 ± 0.02, 0.11 ± 0.03). In D–F, n.s. P > 0.0125, * P < 0.0125, ** P < 0.001, *** P < 10^−7^.

Finally, we asked how body/brain state affects sensory responses in HONs. Given that their activity controls locomotion initiation, which could be critical, for example, for foraging when hungry (Yamanaka et al., 2003), it would be adaptive if fasting increased sensory-evoked HON activation and movement. We food-deprived mice for 24 h and recorded sensory responses (hungry state), then allowed mice to re-feed *ad libitum* for 2 h and recorded again (re-fed state, Fig. 7A). This study design with a relatively short time interval between compared recordings allowed us to limit the effect of other brain state variables such as arousal. This procedure revealed that HON responses to odors were selectively increased by hunger. In particular, responses to food odor and the likelihood of locomotion following food odor were increased (Fig. 7B–D, n = 4 animals). ON and down-up cells increased responses to food odor significantly (P < 0.0125), while up-down cells were the only cell type that increased responses to social odor significantly (Fig. 7E, F). These analyses indicate that food deprivation selectively affects HON odor responses with subtype specificity, suggesting detailed brain state dependent modulation of these responses.

## 3 Discussion

Accumulating literature on LH HONs has focused mainly on slow neuropeptide modulation of long-lasting behavioral states like sleep, arousal, or obesity. This has reinforced a prevailing dogma that the primary function of HONs is a slow coordination of feeding, arousal and metabolism (Sakurai, 2014). However, a few recent studies have begun to show another rapid mode of operation in LH neurons that is causally linked to behavior (Herrera et al., 2016; Jennings et al., 2015; Li et al., 2018; Nieh et al., 2016). Our data now provide strong evidence for a key role of HON activity in subsecond-scale sensorimotor transformations (Fig. 5, Fig. 6), as well as self-initiated movement (Fig. 2, Fig. 3, Fig. 4), and diverse mobile behaviors (Fig. 1). Some previous work has also shown that HONs have rapid sensory responses (González et al., 2016a; Hassani et al., 2016; Inutsuka et al., 2016; Mileykovskiy et al., 2005), but motor impact of these responses remained unclear. Here, we demonstrated that these responses are causally involved in rapid control of spontaneous and sensory-evoked motor output, similar to sensorimotor transformations in the neocortex (Ferezou et al., 2007; Svoboda and Li, 2018), explaining the previously unclear need to update HON sensory representations on a subsecond timescale. In particular, the broad stimulus receptivity (Fig. 5) and robust activation during mobile behaviors (Fig. 1) indicate that HONs have a broad and rapid influence on locomotion initiation.

Based on their activity profiles during self-initiated movement, HONs can be functionally divided into several subclasses. Of the movement-related subtypes, ON cells carried clear predictive information about future movements and were robustly activated when sensory stimuli elicited movement (Figs. 2V and 6 D, E). Up-down cells could also predict sensory-evoked (Fig. 6E) but not self-initiated movement initiation. While none of the subtypes seemed to prefer a particular sensory modality (Fig. 5A–F, and discussion in Results), the modulation of sensory responses was subtype specific: Up-down cells were more uniformly responsive when social odor induced running (Fig. 6D lower panel), and increased social odor responses during hunger while mice were stationary (Fig. 7F). ON cells and down-up cells selectively increased food odor responses during hunger (Fig. 7E). The effect of hunger may reflect an increased HON excitability, consistent with prior *in vitro* observations that reduced glucose levels increase membrane resistance of some HONs thus making their membrane potential more responsive to inputs (Burdakov et al., 2005; Williams et al., 2008); this can be tested by intracellular recordings from HONs *in vivo* in future studies.

Elucidation of the role of the brief HON bursts in different phases of locomotion required a rapid and reversible manipulation of these bursts *in vivo*. We performed these manipulations using optogenetics, and our central conclusions arise from these temporally-targeted acute optogenetic silencing and activation experiments. We also performed temporally-unselective, chronic HON manipulation using the DTR model. As a tool for studying subsecond dynamics of locomotion, the results obtained using the DTR method are expected to be more affected by confounds of compensatory and slow/indirect effects than the optogenetic approach. Indeed, while we have observed locomotion initiation defects using both methods, in the chronic DTR experiments we observed additional alterations such as small effects on bout speed and duration of self-initiated (but not sensory-evoked, Fig. 6K–O) locomotion bouts. These effects may arise due to cardiorespiratory complications of chronic HON deficiency (Kuwaki, 2011; Nattie and Li, 2012), or due to chronic adaptations in self-initiated locomotion such as seen in control mice in this 12 day experiment (Fig. 3J–M).

Our findings relating to rapid motor control are an important advance over previous knowledge of the role of HONs in arousal and motivation. HONs were previously shown to contribute to background control of overall locomotor activity in response to slow manipulations such as fasting (Hu et al., 2015; Takakusaki et al., 2005; Yamanaka et al., 2003). Furthermore, the loss of *tonic* orexin signaling has been linked to motor deficits in cataplexy following HON deletion (Kaushik et al., 2018; Mieda et al., 2004). Although arousal and motivation are very broad terms, which have been used to describe many different sensorimotor states (including wakefulness, attention, movement, goal-oriented behavior, and sexual arousal), previous findings suggested that HONs play a slow, modulatory, role in these processes (Adamantidis et al., 2007; Sakurai, 2014). In contrast, we found that HON activity triggers locomotion on subsecond timescales, consistent with a rapid role in locomotion initiation similar to that of classic motor controllers such as midbrain dopamine neurons (da Silva et al., 2018), rather than a secondary effect of increased attention/arousal. With regard to cataplexy in the absence of a functional hypocretin/orexin system, these findings suggest that the HON-modulation of sensory signals may be essential for maintaining proper muscle tone during unexpected sensory stimulation, pointing at a potential neural basis for cataplectic attacks. Yet, whether and how laughter and other positive emotions, that trigger cataplectic attacks, engage such pathways remains to be investigated.

The labelling of some or all of these results as attention/arousal effects depends on the timescale definition of attention/arousal. The shortest timescale we captured here was 34–324 ms from sensory stimulus to HON activity increase (Fig. 5K). This activity predicted sensory-evoked movement some 600–4600 ms after stimulus onset (Fig. 6E). Exogenous activation of HONs with C1V1 led to movement in 300–4880 ms (Fig. 3F), while internally generated activation of HONs could precede movement by up to 3380 ms (Fig. 5L). While GCaMP6s does not report action potentials immediately as they happen, the temporal resolution of >100 ms is captured reliably, and the overall sequence of sensory/spontaneous HON activation -> EMG activation -> movement is supported strongly by our results. A recent analysis in V1 indicated delays of <10 ms from spike to GCaMP6s fluorescence increase (Huang et al., 2019), and in our settings we estimated 149 ± 30 ms for our multi-cellular resolution imaging at 10 Hz capture rate. For the high speed recording with fiber photometry, this value will depend on the size of the recorded population, and with a large population is likely to correspond to the lowest in the range, which in our hands was 31 ms. It is therefore clear that hypothalamic HONs, despite their paradigmatic position as slow modulators of whole-body physiology, have a rapid effect on motor performance. It is tempting to speculate that HONs send parallel (rather than sequential) outputs to arousal and locomotor systems, for example to ensure that attention and locomotion occur together.

In terms of neurotransmitters and postsynaptic targets that may mediate the rapid effects of HON activity on locomotion initiation, there are several potential candidates. HONs co-release glutamate and orexin onto at least some of their hypothalamic and extrahypothalamic postsynaptic neurons (Blomeley et al., 2018; Schöne et al., 2014), and innervate multiple CNS sites that drive or modulate locomotion, including the substantial nigra, nucleus accumbens, motor cortex, locus coeruleus, and spinal cord (Hagan et al., 1999; Peyron et al., 1998; van den Pol, 1999; Thorpe and Kotz, 2005). How HON firing controls locomotor-related activity at these targets *via* orexin and/or glutamate-induced excitation is a key question for future studies. Glutamate neurotransmission mediated by glutamate-gated AMPA receptors would induce faster postsynaptic excitation than orexin neurotransmission mediated by G-protein coupled receptors (Sakurai et al., 1998; Schöne et al., 2014). However, the role of orexin in the rapid locomotor effects cannot yet be ruled out, since LH activation can produce orexin-receptor-antagonist-sensitive postsynaptic excitation of lumbar spinal motoneurons with <100 ms delays (Yamuy et al., 2004).

Our work places HONs into a growing diagram of movement controlling neurons across the brain (Arber and Costa, 2018; Svoboda and Li, 2018). In particular, HONs initiate locomotion, consistent with ‘stepping’ induced by electrical stimulation of LH in anaesthetized animals (Jordan, 1998; Sinnamon, 1993). A recent study demonstrated similar properties in substantia nigra pars compacta dopamine neurons during self-initiated locomotion; this population had heterogeneous activity profiles during movement with some cells turning off and most being activated, and optogenetic manipulation revealed a highly similar phenotype to what we have shown here for HONs (da Silva et al., 2018). Interestingly, for midbrain dopamine neurons, tonic *vs* burst firing modes have been proposed to have different behavioral roles (Grace, 1991; da Silva et al., 2018), and it would be interesting to investigate whether this occurs in HONs, by future electrophysiological recordings of HON spike discharge. Similar to HONs, classical movement-control neurons in the basal ganglia activate predictably before self-initiated movement (Cui et al., 2013). Thus, we find that HONs closely resemble classical subcortical motor control neurons in the midbrain and striatum. As HONs are in a unique position to affect sympathetic outflow (Bonnavion et al., 2015; Kerman et al., 2007) their pre-locomotor activation could also orchestrate metabolic maintenance of movement, for example, by increasing heart-rate and respiration.

## 4 Methods

### 4.1 Animals

Animal handling and experimentation was approved by the UK government (Home Office) and by Institutional Animal Welfare Ethical Review Panel. Animals of both sexes, aged 30–60 days at the beginning of the procedures were used and were housed in a controlled environment on a reversed 12 h light-dark cycle with food and water *ad libitum*. WT C57BL6 mice were obtained originally from the Jackson Laboratory. Mice expressing the human diphtheria toxin receptor in HONs (O-DTR mice) were generated as described before (González et al., 2016b) and cross bred with WT mice. The specific deletion of HONs with DT in this strain has been documented before (González et al., 2016b) and was confirmed in the experimental animals in this study (see Fig. 3H). O-DTR mice had 3.6 ± 1.8 % HONs remaining compared to WT mice after DT (cell counts from 6 mice, 3 bilateral sections from each mouse: 2268 HONs in WT DT + and 82 in O-DTR DT+). For fiber photometry experiments in Fig. 5J–L, orexin-cre mice (Schöne et al., 2012) were used.

### 4.2 Surgery: virus injections

Two to three weeks prior to optical device implantation, WT mice were injected stereotactically with AAV1-hORX.GCaMP6s (prepared by Penn vector core, PA, USA), AAV1-hORX.C1V1(t/s).mCherry or AAV1-hORX.ArchT.TdTomato (prepared by Vigene Biosciences, MD, USA). The GCaMP6s virus was generated as described before (González et al., 2016b) using the human prepro-orexin promoter sequence (Sakurai et al., 1999) kindly donated by Dr Takeshi Sakurai, which has previously been used to generate a widely used orexin-eGFP mouse line (Burdakov et al., 2006). Our other two virus constructs were cloned using the same hORX.GCaMP6s plasmid for the prepro-orexin promoter and plasmids acquired from Addgene for the opsin and fluorescent protein sequences. Using the orx.GCaMP6s virus, GCaMP6s was expressed with 97.4 ± 1.0 % specificity and 65.8 ± 3.7 % penetrance in HONs (381 ± 73 orexin+, 242 ± 40 GCaMP6s/orexin + and 6 ± 2 GCaMP6s/orexin- cells counted from 4 animals, Fig. 1G). The mean delay from onset of a spiking train to detected fluorescence increase was 149 ± 30 ms (n = 6 cells, range 31–288 ms, Fig. 1D). For experiments in Fig. 5J–L, orexin-cre mice were injected in the left hemispheres with AAV9-CAG.Flex.GCaMP6s.WPRE.SV40 (prepared by Penn vector core, PA, USA) and expression has been characterized before (González et al., 2016a, 2016b).

Using the orx.C1V1.mCherry virus, C1V1.mCherry was expressed with 96.0 ± 0.2 % specificity and 54.8 ± 3.4 % penetrance in HONs (224 ± 36 orexin+, 119 ± 13 mCherry/orexin + and 6 ± 3 mCherry/orexin- cells counted from 4 animals, Fig. 3B). Using the orx.ArchT.TdTomato virus, ArchT.TdTomato was expressed with 99.1 ± 0.4 % specificity and 59.1 ± 3.3 % penetrance in HONs (289 ± 92 orexin+, 161 ± 38 TdTomato/orexin + and 1 ± 1 TdTomato/orexin- cells counted from 5 animals, Fig. 4B). For surgery, mice were anesthetized with isoflurane, the scalp was infiltrated with lidocaine, opened, and a 0.2 mm craniotomy was drilled at 0.9 mm lateral, 1.4 mm posterior from Bregma (in the right hemisphere for imaging experiments and bilaterally for optogenetic manipulation and control experiments). A pulled glass injection needle was used to inject 100−400 nl of virus 5.4 mm deep in the brain at a rate of 50 nl/min. After removal of the injection needle, the scalp was sutured and animals received 5 mg/kg carprofen injections for two days as post-operative pain medication.

### 4.3 Surgery: optical device implantation

Two weeks after viral delivery mice were anaesthetized with isoflurane, the scalp infiltrated with lidocaine, and opened. A custom-made aluminium head plate was attached to the skull using three skull screws and dental cement (Metabond). A 0.8 mm diameter hole was drilled at the same position(s) as in virus injection surgery and the optical device was slowly (150 μm/min) lowered to a depth of 5.1 mm using an automated Luigs & Neumann micromanipulator controlled from Matlab. Lens placements in Fig. S2. For imaging experiments, the optical device was a 0.39 NA, 7.3 mm long, 0.6 mm diameter cylindrical graded refractive index (GRIN) lens (Grintech). For fiber photometry experiments in Fig. 5J–L, the optical device was a 200 μm core fiber optic cannula (Doric MFC_200/260-0.37_50mm_MF2.5(7.5 mm)_FLT, or ThorLabs CFML12U-20, NA 0.39). For optogenetic experiments (and GCaMP controls in optogenetic experiments), the optical device consisted of two 0.39 NA, 10 mm long, 0.2 mm diameter optic fiber cannulae. After lowering optical devices into the LH, they were cemented onto the skull and the implant was coated with black dental cement and painted with 3–4 layers of black nail polish to keep scattered light from entering the environment.

The mice received a single dose of 0.6 mg/kg dexamethasone and 5 mg/kg carprofen injections for two days as post-operative pain and anti-inflammatory medication. After at least two weeks of recovery, mice were trained for head-fixed awake experiments in 5–10 sessions of increasing length on the experimental running-wheel apparatus or for freely moving miniature endoscope recordings in a similar incremental regimen of wearing the head-mounted miniature microscope (∼2 g weight) in the test arena (Fig. 1E).

### 4.4 Freely moving Ca^2+^ imaging

Freely moving Ca^2+^ imaging was performed with a head-mounted miniature microscope (Inscopix) which was attached to the animal’s head without anaesthesia to a magnetic baseplate that had previously been cemented at the correct focal height on top of the implant. The subject was then placed into the plexiglass-floored arena which it had already spent time in (during many hours of habituation sessions with the miniature microscope) and recording was begun after 10 min. A typical recording session lasted 30 min during which time a familiar opposite-sex conspecific was introduced by a gloved handler (conspecific was supported on palm rather than held by tail during entry), and sometimes (rarely) the handler’s gloved hand would ‘chase’ the subject. Most of each recording was simply observation of the subject and conspecific in a familiar arena with wet food available *ad libitum*. The arena was lit with red LED lighting and was covered from view in a dark, quiet room and a silent fume extractor pipe was located 50 cm above the arena to eliminate background odors. Ca^2+^ imaging was performed at a frame rate of 10 frames/s. Behavior tracking was done with a CCD camera (Lumenera Infinity) below the arena acquiring at 60 frames/s and synchronized with Calcium imaging using an LED mounted next to the arena and facing the behavior camera. The LED was driven to flash on every acquired Calcium imaging frame with a TTL pulse from the microscope DAC board. Behavior analysis was done in ImageJ by manually classifying behaviors and measuring distance and time travelled during each behavior bout.

### 4.5 Head-fixed two-photon Ca^2+^ imaging

Mice were imaged for 0.5–1 h head-fixed on a running disk allowing the animal to move or remain stationary *ad libitum*. Each mouse was recorded in 2–8 sessions to obtain the full datasets presented here, and cell numbers from each mouse (26–85) were stable across recording sessions (typical variance <15.5 %). Imaging was performed either in a dark room with red background lighting (Fig. 2, Fig. 5) or with a 21-inch flat screen monitor located 15 cm away from the animal’s eyes and displaying a low luminosity picture of a natural forest scene (Fig. 6). Throughout imaging sessions, we recorded the movement of the running disk *via* a custom-built infra-red optical sensor which was sensitive to small movements and encoded speed linearly as the differential of the optical signal. Locomotion epochs were defined as the time when the mouse moved >1 cm/s. A breathing sensor was placed near the right nostril to monitor normal breathing.

Changes in GCaMP6s fluorescence were imaged with a custom electro-tunable lens equipped resonant/galvanometer scan head two-photon microscope (INSS) and a femtosecond-pulsed mode-locked Ti:sapphire laser (Spectra-physics Mai Tai HP Deepsee 2) at 950 nm through a 20 × (0.45 NA, Olympus) air-IR objective at 31frames/s with 512 × 512 pixel resolution using custom Labview software. With 5-plane imaging using the electro-tunable lens volume rate was 5.1 volumes/s. Images were obtained with a 510/80 nm band-pass emission filter.

Visual stimuli were delivered with a blue LED positioned at eye level 10 cm away and visible to both eyes. Tactile stimuli were delivered with a fast TTL driven valve device (picospritzer) through a through a fan shaped nozzle (15 mm length, 3 mm width, which formed a air stream approximating a fan with 60° angle) so that the geometry of the airpuff impinging on the animal was insensitive to small postural changes. Tactile stimuli were either 0.2 s pulses of 30 psi pressure delivered to the left side lower abdomen from the side as a vertical fan pattern (Fig. 5) or 2 s pulses of 15 psi pressure delivered to the base of the tail from the back as a horizontal fan pattern (Fig. 6). Olfactory stimuli were delivered with a custom TTL driven solenoid valve switching device, covered in a sound-proof case, which delivered a constant 1 psi stream of air through an empty vial to the animal’s snout from 1 cm distance. The device created odor pulses by switching valves rapidly to direct the air through an odor vial containing amylacetate, female urine (∼0.5–1 ml collected fresh from 5 separately housed female mice), ethylbutyrate, acetophenol or eugenol. Odor pulses and background odors were cleared by a constant-suction high-capacity fume extrusion pipe (8 cm diameter) located 5 cm in front of the animal which was there during all recordings. Odor delivery and lack of pressure changes was confirmed by the experimenter. The null stimulus in Fig. 5 was an auditory stimulus delivered through speakers located 15 cm in front of the animal and driven by a 10 kHz pure tone clip played from Matlab using the psychophysics tool box. The auditory stimulus intensity was 77 dB and background noise on the microscope was 65 dB, and it did not generate sensory responses in HONs.

The presentation of sensory stimuli was synchronized with image acquisition using custom routines in Matlab to generate timing triggers. Within Matlab, a National Instruments USB-6008 DAQ board was used to output TTL triggers and count imaging frames. Stimuli were presented in blocks consisting of a visual, tactile, auditory and 1–3 odors presented for 0.2 s (Fig. 5) or tactile and an female urine odor presented for 2 s and 0.5 s respectively (Fig. 6). Stimulus interval was 15 s plus a randomly selected 0.1−15 s re-randomized before every stimulus presentation (Fig. 5) or 30 s plus a randomly selected 0.1–30 s (Fig. 6). In addition, order of the stimuli was randomized for each block. Before every experiment mice were allowed to habituate to head-fixation and stimulus presentation for 5–10 min.

### 4.6 Head-fixed Ca^2+^ fiber photometry

GCaMP6s was excited with a blue LED (Prizmatix UHP-LED-460, 460 nm, ThorLabs M470F3, 470 nm, Fig. 5M) or a 473 nm laser (Becker & Hickl, Fig. 5J–L) and emission was sampled at 500 Hz with a photoreceiver (Newport 2151 or Becker & Hickl HPM-CON-2) through a fiber-COnnectorized GFP filter cube (Doric, FMC_GFP_FC). Optic fibers were from ThorLabs (multimodal, FT200EMT, 200 μm core diameter, 0.39 NA) or Doric (MFP_200/230/900-0.22_2m_FCM-MF). The subjects were on a treadmill consisting of a disk with a rotary encoder that pulsed 24 times per rotation. The mice were placed at about 7 cm from the center of the wheel. Thus, the distance walked with each tick was approximately 1.83 cm. As this could increase variability of the estimated run start time, we averaged 11–28 running bouts from each animal. Sensory stimuli were delivered as in two photon imaging experiments.

### 4.7 Calcium imaging analysis

Initial image processing including correcting motion artifacts and drift in the imaging plane were done using the TurboReg plug-in in ImageJ (NIH) for 2-photon data or Mosaic (Inscopix) for miniature endoscope data. Imaging data were down-sampled spatially by 2*2 (2-photon) or 3*3 (miniature endoscope) binning to reduce file size. Cell outlines were drawn manually, and, in head-fixed 3-D 5-plane imaging, outlines containing the same cell were tracked across planes. Mean intensity within each region of interest (ROI) was used to generate *F_raw_*. The local neuropil signal was estimated from a neuropil mask containing the third to the sixth nearest pixels outside the outline that did not contain other ROIs, which was used to generate a mean neuropil signal *F_np_*. ROI specific signals were calculated as *F_raw_* –*F_np_*. For 2-photon imaging the ROI signals in neighbouring planes that clearly originated from the same cell were averaged to get the cell specific signal. The ROI specific signal was corrected for photobleaching in miniature endoscope data by computing ΔF/F_0_ using a median over a 100 s moving window as F_0_, to get the cell specific signal. Cell specific signals were Z-scored for plotting and analysis. In Fig. 7, hungry and re-fed imaging sessions were concatenated for Z-scoring so they could be compared reliably.

To define cell types, noise was reduced by smoothing *F_raw_* and *F_np_* with a 3 frame moving average, then average activity for each cell specific signal was calculated from bouts aligned to the onset of locomotion (Fig. 2C). Baseline and 2 s.d. threshold were calculated from 10 – 6 s before locomotion onset. Extrema were found during the period 6 s before to 16 s after locomotion onset, and minimum and maximum responses were computed as 1 s averages around the extrema. If a cell had only a maximum above the 2 s.d. threshold from baseline it was called an ON cell, if it only had a minimum below 2 s.d. from baseline it was an OFF cell, if both of the previous conditions were true the cell was either an up-down or down-up cell (depending on the sequence of the extrema), and otherwise it was called not modulated. Onset times relative to movement onset (Fig. 2P, Q) were calculated as the point where cell specific signal deviated at least 1 s.d. and stayed for at least 2 s at least 1 s.d. deviated from baseline. To calculate fractions in Fig. 2R, S, we used a cutoff of mean +2 s.d. from the whole recording for each cell to define active frames. Also, as locomotion-related activity preceded and followed locomotion epochs by some seconds, we defined movement frames for this analysis as those during movement and 3 s before and after.

In Fig. 2T ON and up-down cells with the longest onset lead time were selected from previous imaging data and their average activity in each frame and the two preceding frames is plotted. In Fig. 2U and V, a linear support vector machine (SVM) classifier with a fixed regulation parameter C = 1 was trained on the population activity vectors from an equal amount of locomotion and stationary epochs (drawn at random from intervals between locomotion bouts, such that no locomotion occurred during or within 5 s of these epochs) using LIBSVM (Chang and Lin, 2011). There were between 8–30 epochs of each kind/ animal, and 6 animals in this analysis. For each animal, the classifier was trained on randomly drawn 87.5 % of the data. It then estimated a decision boundary, which was used to classify the vectors from the remaining 12.5 % of the data. The classification process was repeated using a 8-fold cross-validation procedure for which another (non-overlapping) 12.5 % of the data served as test-data while the classifier was again trained on the other 87.5 % of the data, until all of the 12.5 % proportions were independently used for testing once. Because it was still possible that the classification process could be influenced by a drawing bias, all eight cross-validation steps were additionally repeated ten times with newly drawn 12.5 % proportions for testing, resulting in a total of 80 analyses, which were averaged to obtain the classification accuracy for each time window. This cross-validation procedure is very conservative, and given the noisiness of Ca data in general, only very stable differences between activity patterns would lead to significant results. We modified code from DDTBOX (Bode et al., 2019) toolbox to do these analyses in Matlab.

Fiber photometry data in Fig. 5J–L was corrected for photobleaching by computing ΔF/F_0_ using a mean over a 2 s period before each stimulus as F_0_, followed by Z-scoring. Fiber photometry data in Fig. 5M was corrected for photobleaching by computing ΔF/F_0_ using a mean over a 240 s moving window as F_0_, followed by Z-scoring. The signal was either averaged across sensory trials and smoothed with a 5 sample sliding average (Fig. 5J–L), or averaged across locomotion onsets and smoothed with a 50 sample sliding average (Fig. 5M). Signal onset was defined as first frame where signal increased 1 s.d. above baseline (10−6 s before locomotion, or 2.5–0 s before sensory stimulus) for at least 0.2 s.

### 4.8 Optogenetics and O-DTR ablation experiments

Optogenetic manipulations were performed in the same head-fixed apparatus that was used for 2-photon imaging. Two 532 nm green lasers (Laserglow) were used to deliver light through shielded 0.2 mm diameter optic fibers to the bilateral implants. Light intensity out of the fiber was set to 3 mW for ArchT stimulation (constant on for 10, 12.05 or 30 s as indicated) and 20 mW for C1V1 stimulation (10 ms pulses for 2.5 s at indicated frequency). HONs in O-DTR mice were deleted by two injections of 150 ng diphtheria toxin (Sigma D0564) diluted to 1 μg/ml in saline, administered i.p. on day 0 and day 2. Recordings for DT + conditions were started on day 12. WT control mice were treated identically in parallel. In experiments in Fig. 4, Fig. 6F–O, the trial would not start until the mouse was stationary for at least 5 s. In addition, trials were spaced by 15 s + randomized 0.1–10 s intervals (Fig. 4) or by 25 s + randomized 0.1–25 s intervals (Fig. 6F–O) and the order of laser-on and laser-off (catch) trials was randomized in each block (consisting of one of each trial). In Fig. 6F–J laser was turned on 50 ms before stimulus presentation. In order to limit the total duration of each recording session, due to the number of stimuli and length of intervals, the datasets for ArchT and O-DTR experiments were combined across two recording sessions from each mouse. As HON deletion can lead to a cataplectic phenotype, we monitored video footage of the mice during locomotion recordings to check for signs of abrupt sleep attacks or loss of muscle tone. However, no atonia was seen throughout recordings in the O-DTR DT + and ArchT laser + conditions, and in the ArchT experiments, there was no effect on speed or duration of movement bouts, suggesting strongly that cataplectic attacks did not occur in these recordings. This is also consistent with the rarity of cataplectic attacks in the absence emotional triggers (Hara et al., 2001; Scammell et al., 2009), and a previously demonstrated lack of effect of transient orexin-ArchT inhibition during the dark phase (Tsunematsu et al., 2013). Care was taken to record each mouse at the same time of day to minimize circadian effects. Before every experiment mice were allowed to habituate to head-fixation and stimulus presentation and or optical manipulation for 5−10 min.

### 4.9 Preparation of acute slices

Coronal brain slices from P60-180 animals were prepared after instant cervical dislocation and decapitation. The brain was rapidly dissected and cooled in continuously gassed (95 % O_2_ and 5% CO_2_), icy cutting solution containing (in mM): 90 *N*-methyl-d-glucamine, 20 HEPES, 110 HCl, 3 KCl, 10 MgCl_2_, 0.5 CaCl_2_, 1.1 NaH_2_PO_4_, 25 NaHCO_3_, 3 pyruvic acid, 10 ascorbic acid and 25 d-glucose. 350 μm thick coronal brain slices were cut on a vibratome (Campden) and allowed to recover for 5–15 min at 37 °C in cutting solution, followed by 45–55 min at 22 °C in artificial cerebrospinal fluid (ACSF) containing (in mM): 126 NaCl, 3 KCl, 2 MgSO_4_, 2 CaCl_2_, 1.1 NaH_2_PO_4_, 26 NaHCO_3_, 0.1 pyruvic acid, 0.5 l-glutamine, 0.4 ascorbic acid and 25 d-glucose, continuously gassed with 95 % O_2_ and 5% CO_2_.

### 4.10 Slice electrophysiology

Patch clamp recordings were performed in a submerged chamber with 3−5 ml/min superfusion with ACSF, continuously gassed with 95 % O_2_ and 5% CO_2_. 3–6 MOhm patch pipettes were filled with intracellular solution containing (in mM): 130 K-gluconate, 5 NaCl, 2 MgSO_2_, 10 HEPES, 0.1 EGTA, 4 Mg-ATP, 0.4 Na-GTP, 2 pyruvic acid, 0.1 Alexa-594, 0.1 % biocytin, and ∼10 mM KOH (to set pH to 7.3). Whole cell recordings were not analysed if the access resistance was above 25 MOhm. Voltage recordings were sampled at 10 kHz and low-pass filtered at 3 kHz with HEKA EPC10 usb amplifiers and acquired with HEKA patchmaster software. ArchT and C1V1 were stimulated with green light from a xenon lamp (Sutter lambda 4DG controlled from HEKA patchmaster) through a TRITC-filter. Concurrent GCaMP6s fluorescence recordings were performed using the miniature endoscope with a GRIN lens implant recovered from a successful *in vivo* recording. This assembly was mounted on a micromanipulator and moved into focus at about 350 μm away from the patch clamped cell. Patch clamp data were analyzed in Matlab.

### 4.11 Immunohistochemistry

Mice were overdosed with ketamine/xylazine (100 mg/kg and 10 mg/kg respectively) and transcardially perfused with 4% PFA. Brains were post-fixed for 24 h. Coronal brain slices were sectioned at 50 μm using a cryostat or a vibratome. Sections were blocked in PBS with 0.3 % Triton X-100 and 1% bovine serum albumin (blocking solution) for 1 h, incubated with goat anti-orexin (1:1000; Santa Cruz) over-night, washed, incubated with Alexa 647 conjugated donkey anti-goat (1:1000; Invitrogen) for 3.5 h, washed and mounted. Antibodies were applied blocking solution. Confocal micrographs were acquired on an Olympus FV1000 and merged in imageJ.

### 4.12 Statistics

All data are shown as mean ± SEM unless stated otherwise. Statistical significance was determined by paired Student *t*-test or Wilcoxon rank sum test as stated. All statistics were performed using statistical functions in Matlab. Sigmoid fits in Fig. 3C and E are based on modified Hill equations $S=\frac{S_{max}*F^{h}}{{EC}_{50}^{h}+F^{h}}$ (Fig. 3C) or $P=P_{max}-\frac{P_{max}*F^{h}}{{IC}_{50}^{h}+F^{h}}$ (Fig. 3E), where S is spike output, F is light pulse frequency, P is movement probability, EC_50_ is half-maximal response and IC_50_ half-maximal decrease. Fitting was done using custom routines in Matlab.

## CRediT authorship contribution statement

**Mahesh M. Karnani:** Conceptualization, Data curation, Formal analysis, Funding acquisition, Investigation, Methodology, Project administration, Supervision, Software, Validation, Visualization, Writing - original draft, Writing - review & editing. **Cornelia Schöne:** Formal analysis, Investigation, Methodology, Writing - review & editing. **Edward F. Bracey:** Investigation, Methodology, Writing - review & editing. **J. Antonio González:** Investigation, Methodology, Writing - review & editing. **Paulius Viskaitis:** Investigation, Methodology. **Han-Tao Li:** Investigation, Methodology. **Antoine Adamantidis:** Conceptualization, Methodology, Writing - review & editing. **Denis Burdakov:** Conceptualization, Funding acquisition, Methodology, Project administration, Resources, Writing - original draft, Writing - review & editing.

## Declaration of Competing Interest

The authors declare no competing interests.
