# Supplementary material for: Role of spontaneous and sensory orexin network dynamics in rapid locomotion initiation
Source: Prog Neurobiol. 2020 Apr;187:101771. doi: 10.1016/j.pneurobio.2020.101771 (PMC7086232; doi:10.1016/j.pneurobio.2020.101771)
Supplement: Supplementary file 1 [file mmc1.pdf]

Supplemental Information titles and legends

Movie M1.

3D 2-photon imaging of HONs in vivo.

This is a 26 minute movie, sped up 60x such that each second corresponds to one minute in real time. Top panel shows all planes colour coded and merged together, and middle panels show each of the 5 planes imaged sequentially at 5.1 Hz. Bottom panel shows the fluorescence traces from green, numbered regions of interest within the panels. Purple circles are other cells drawn in to guide the eye.

Figure S1.

Characterization of orx.GCaMP6s virus expression.

A, Schematic of sample preparation. B, An HON in a brain slice was imaged with a miniature endoscope at an angle (above micrographs: AVG, average time projection; STD, standard deviation projection; recorded neuron at arrow head) and with patch clamp microscope from above (below left, GCaMP6s fluorescence; below right, merged GCaMP6s, Alexa-568 red patch pipette filling solution and oblique illumination micrograph showing patch pipette; recorded neuron at arrow head). C, Ca^{2+}-imaging (green, Z-scored $\Delta F/F_0$), electrophysiology (black) and firing rate (red) example data from cell in B. D, Expanded view of firing burst onset showing short latency from firing burst to Ca^{2+} increase. E, Combined data across 6 recordings (each recording in a different colour) showing linear relation between maximal fluorescence and firing rate during each 10s current step. R^2 of linear fit is 0.91. F, Representative micrographs showing orexin immuno-reactivity (IR) and GCaMP6s in coronal sections prepared from an experimental subject. GRIN lens track is visible dorsal to the HONs.

Figure S2.

GRIN lens placement.

A, Coronal sections from two mice illustrating GRIN lens placement in LH (arrow). B, Confocal micrograph of HONs and GRIN lens track (arrow) in coronal section. C, Reconstructed approximate imaging plane locations in the 9 mice used in this study. Blue = 2 mice that were recorded only with miniature endoscope in the freely-moving paradigm, red = 4 mice that were recorded only with 2-photon microscopy in head-fixed paradigms, green = 3 mice that were recorded in both paradigms.

Figure S3.

Distribution of HON locomotion subtype somata in the imaged volume.

Soma centroids locations were computed from imaging volumes and the maps from 7 mice were overlayed and cell locations were labelled by locomotion subtype (as in Fig2 and Fig5-7).

A1-3, scatterplots from various angles showing all 329 cells. B1-3 mean ± s.d. within each population plotted within the volume seen from same angle as the associated scatter plot. ON

and down-up cells had a slight tendency to be more posterior than up-down cells. Axes units are µm.

Figure S4.

Orexin fiber photometry and EMG recording.

A, Example recording of neck muscle EMG spectrogram (above) and broadband power (below blue, cyan is smoothed trace), with simultaneous fiber photometry fluorescence from HONs (black) in LH expressing orx.GCaMP6s virus. Green vertical lines are analysis time points that were averaged for plots in B. B, average fluorescence (black, magenta is smoothed trace for clarity) across 56 (mouse 1, left) or 42 (mouse 2, right) epochs where EMG power increased from baseline as shown in A. Mean EMG broadband power also plotted in blue (cyan is smoothed trace for clarity). HON activity increased 0.84 or 0.76 s before onset of muscle activity.

Supplemental Information for

Rapid sensory integration in HONs governs probability of future movements

Mahesh M. Karnani, Cornelia Schöne, Edward F. Bracey, J. Antonio González, Paulius Viskaitis, Han-Tao Li, Antoine Adamantidis & Denis Burdakov

Correspondence to: mahesh.karnani@hest.ethz.ch

Supplement includes:

Figures S1 to S4
Movie M1

Movie M1.

3D 2-photon imaging of HONs in vivo.
This is a 26 minute movie, sped up 60x such that each second corresponds to one minute in real time. Top panel shows all planes colour coded and merged together, and middle panels show each of the 5 planes imaged sequentially at 5.1 Hz. Bottom panel shows the fluorescence traces from green, numbered regions of interest within the panels. Purple circles are other cells drawn in to guide the eye.

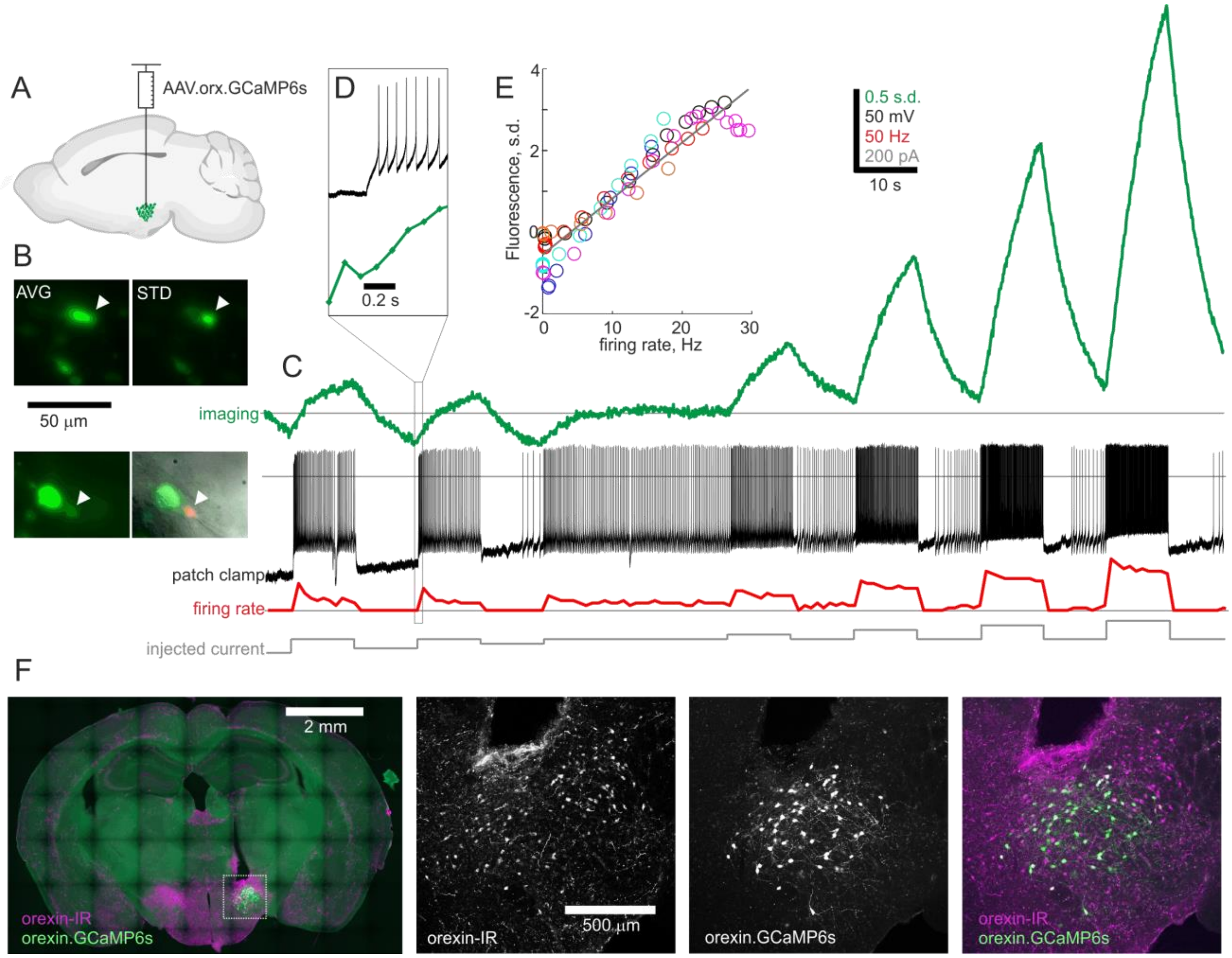

Figure S1.

Characterization of orx.GCaMP6s virus expression.
A, Schematic of sample preparation. B, An HON in a brain slice was imaged with a miniature endoscope at an angle (above micrographs: AVG, average time projection; STD, standard deviation projection; recorded neuron at arrow head) and with patch clamp microscope from above (below left, GCaMP6s fluorescence; below right, merged GCaMP6s, Alexa-568 red patch pipette filling solution and oblique illumination micrograph showing patch pipette; recorded neuron at arrow head). C, Ca^{2+}-imaging (green, Z-scored $\Delta F/F_0$), electrophysiology (black) and firing rate (red) example data from cell in B. D, Expanded view of firing burst onset showing short latency from firing burst to Ca^{2+} increase. E, Combined data across 6 recordings (each recording in a different colour) showing linear relation between maximal fluorescence and firing rate during each 10s current step. R^2 of linear fit is 0.91. F, Representative micrographs showing orexin immuno-reactivity (IR) and GCaMP6s in coronal sections prepared from an experimental subject. GRIN lens track is visible dorsal to the HONs.

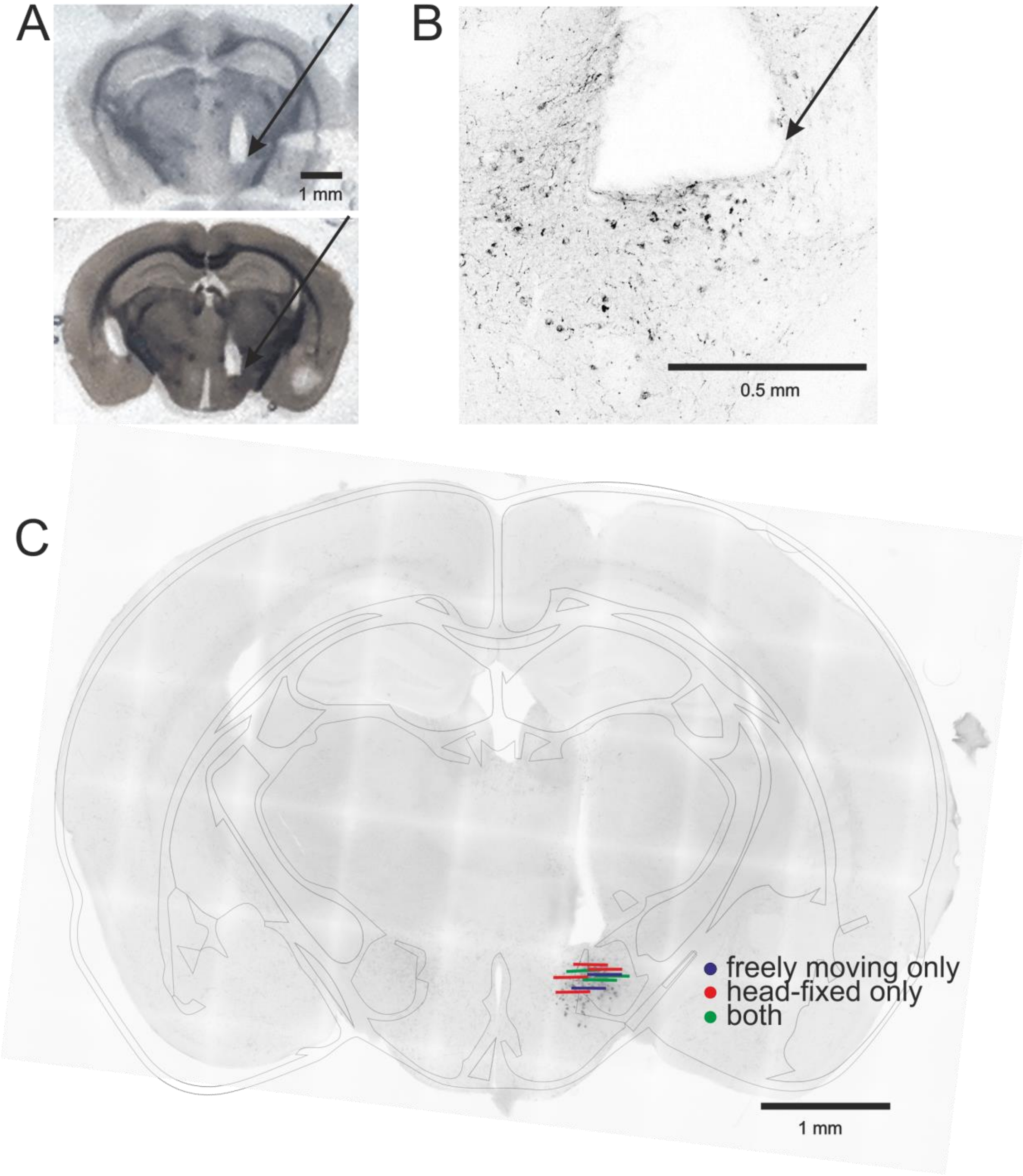

Figure S2.

GRIN lens placement.

A, Coronal sections from two mice illustrating GRIN lens placement in LH (arrow). B, Confocal micrograph of HONs and GRIN lens track (arrow) in coronal section. C, Reconstructed approximate imaging plane locations in the 9 mice used in this study. Blue = 2 mice that were recorded only with miniature endoscope in the freely-moving paradigm, red = 4 mice that were recorded only with 2-photon microscopy in head-fixed paradigms, green = 3 mice that were recorded in both paradigms.

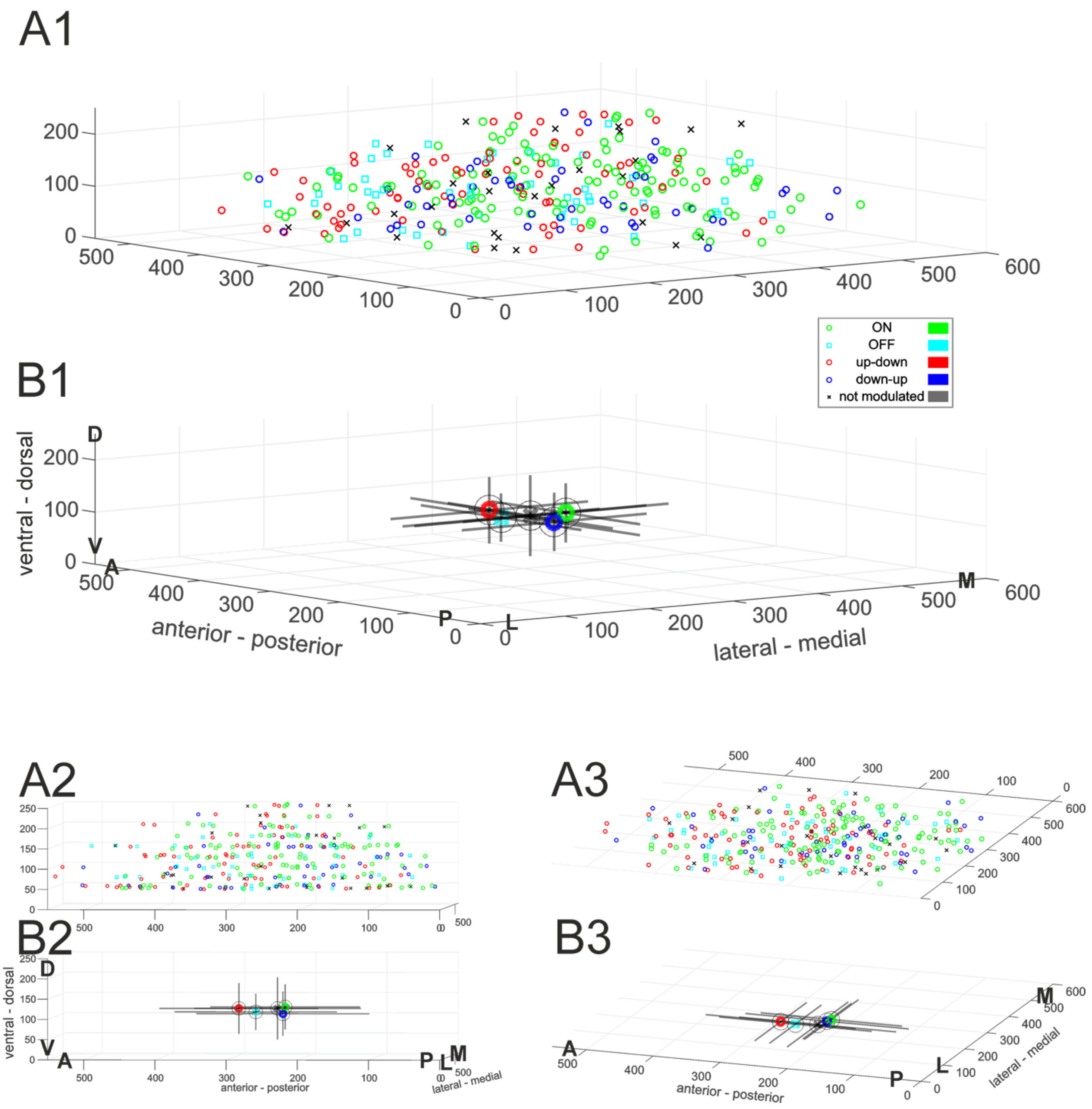

Figure S3.

Distribution of HON locomotion subtype somata in the imaged volume.

Soma centroids locations were computed from imaging volumes and the maps from 7 mice were overlayed and cell locations were labelled by locomotion subtype (as in Fig2 and Fig5-7).

A1-3, scatterplots from various angles showing all 329 cells. B1-3 mean ± s.d. within each population plotted within the volume seen from same angle as the associated scatter plot. ON and down-up cells had a slight tendency to be more posterior than up-down cells. Axes units are µm.

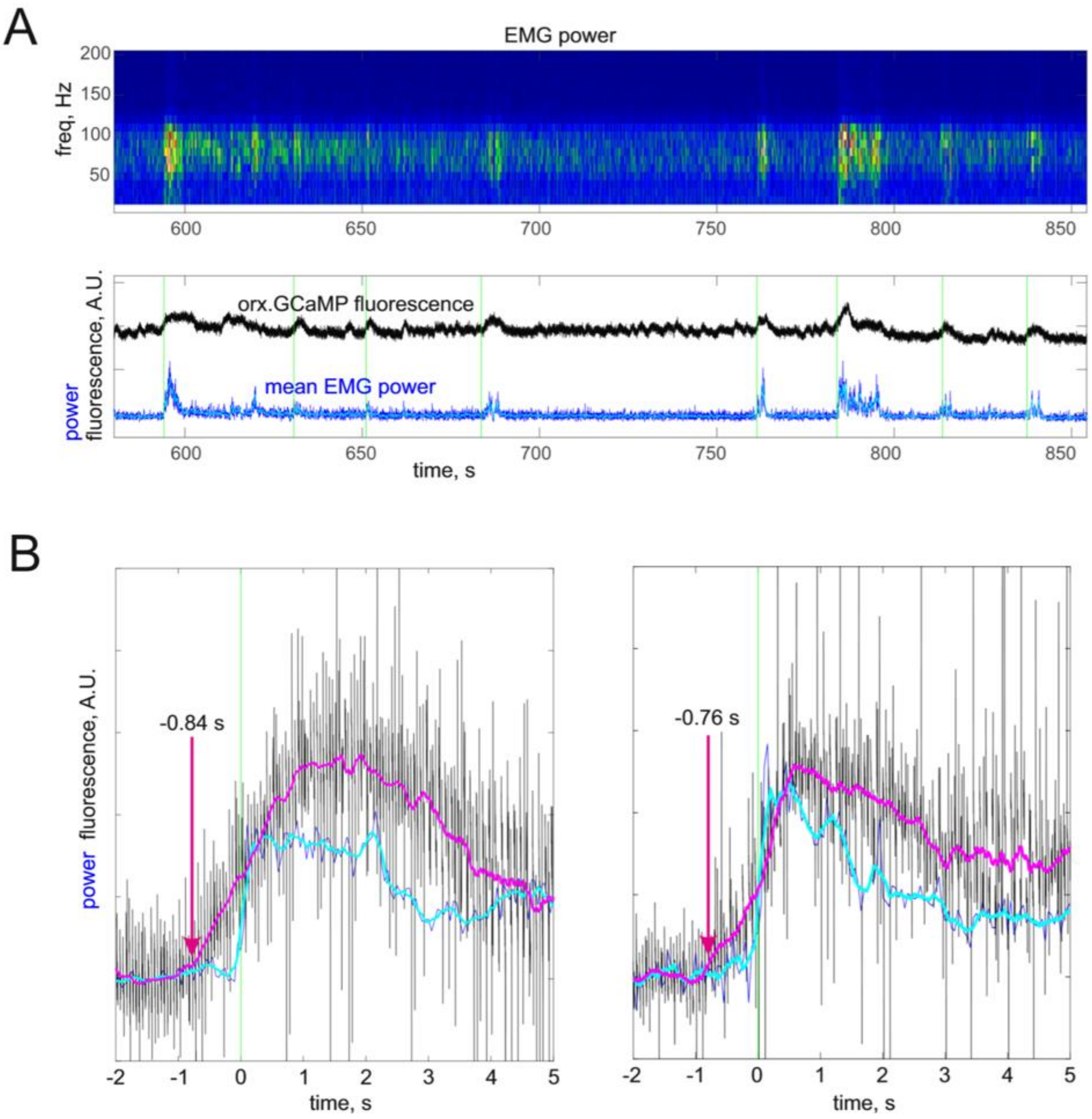

Figure S4.

Orexin fiber photometry and EMG recording.

A, Example recording of neck muscle EMG spectrogram (above) and broadband power (below blue, cyan is smoothed trace), with simultaneous fiber photometry fluorescence from HONs (black) in LH expressing orx.GCaMP6s virus. Green vertical lines are analysis time points that were averaged for plots in B. B, average fluorescence (black, magenta is smoothed trace for clarity) across 56 (mouse 1, left) or 42 (mouse 2, right) epochs where EMG power increased from baseline as shown in A. Mean EMG broadband power also plotted in blue (cyan is smoothed trace for clarity). HON activity increased 0.84 or 0.76 s before onset of muscle activity.
