# Supplementary material for: Role of spontaneous and sensory orexin network dynamics in rapid locomotion initiation
Source: Prog Neurobiol. 2020 Apr;187:101771. doi: 10.1016/j.pneurobio.2020.101771 (PMC7086232; doi:10.1016/j.pneurobio.2020.101771)
Supplement: Supplementary file 2 [file mmc2.pdf]

Peer Review Overview

Manuscript Title: Role of spontaneous and sensory orexin network dynamics in rapid locomotion initiation

Received	03-Jan-2020
1st Decision	31-Jan-2020
Revision Submitted	31-Jan2020
Accepted	03-Feb-2020

Note from the Editor:

This manuscript was transferred from another journal. The review comments and author response letters from this previous submission are not included. Therefore, this peer review overview does not span the complete conversation between the authors and reviewers.

Decision Letter

Ref.: Ms. No. PRONEU-D-20-00002
Role of spontaneous and sensory orexin network dynamics in rapid locomotion initiation
Progress in Neurobiology

Dear Dr. Karnani,

Thank you for submitting your manuscript to Progress in Neurobiology. We have received comments from one previous reviewer on your manuscript. As you will see, the reviewer makes some suggestions regarding the discussion, which are at your discretion for consideration with the final manuscript. We wanted to give you a final opportunity to consider the suggestions before final acceptance.

When resubmitting your manuscript, please carefully consider all issues mentioned in the reviewers' comments, outline every change made point by point, and provide suitable rebuttals for any comments not addressed.

To submit your revised manuscript go to https://www.editorialmanager.com/proneu/ and log in as an Author where you will see a menu item called 'Submission Needing Revision'.

Please resubmit your manuscript by Mar 31, 2020.

We look forward to receiving your revised manuscript.

Kind regards,

Sabine Kastner
Editor-in-Chief
Progress in Neurobiology

Comments from the Editors and Reviewers

Reviewer 1:

Following revisions to this manuscript previously submitted to and transferred from [the previous journal], the authors have satisfactorily addressed all of the comments by the reviewers and further enhanced the already excellent manuscript. By application of multiple cutting edge techniques, the authors have brought out in fine detail the activity and role of the HON neurons in locomotion and sensory-motor function. The research thus makes a substantial contribution to the understanding of HON neuron modulation of behavior and state. My only remaining question which the authors addressed in a minimal manner is why they do not choose in the Discussion to make a greater link between their demonstrated role of HON neurons in sensory-motor responses and the (often sensory induced or dependent) cataplexy that occurs in narcoleptic patients in the absence of hypocretin-orexin. However, this is their choice may be as they point out an issue that could be examined in the future. Its cursory consideration does not detract from an otherwise excellent Discussion and manuscript.

Author Response Letter

Reviewer 1:

Response: We added rows 389-394 in Discussion to make a more pronounced link to cataplexy. Thank you for this added input.
